# GAE-YOLO: a lightweight multimodal detection framework for tomato smart agriculture with edge computing

**DOI:** 10.3389/fpls.2025.1712432

**Published:** 2025-11-19

**Authors:** Xiaoke Liu, Wenjie Teng, Haoran Yu, Zhuoyi Yao, Chengzhen Wang, Yuzhong Peng, Xiaoqing Han, Jianming Liu

**Affiliations:** 1 School of Basic Medical Sciences, Shandong Second Medical University, Weifang, Shandong, China; 2 Weifang Key Laboratory of Collaborative Innovation of Intelligent Diagnosis and Treatment and Molecular Diseases, School of Basic Medical Sciences, Shandong Second Medical University, Weifang, Shandong, China; 3 School of Public Health, Shandong Second Medical University, Weifang, Shandong, China

**Keywords:** tomato smart agriculture, lightweight YOLO, edge computing, multimodal detection, plant phenotyping

## Abstract

**Introduction:**

The advancement of smart agriculture has witnessed increasing applications of computer vision in crop monitoring and management. However, existing approaches remain challenged by high computational complexity, limited real-time capability, and poor multi-task coordination in tomato cultivation scenarios.

**Methods:**

To address these limitations, an intelligent tomato management system is proposed based on the Ghost-based Adaptive Efficient You Only Look Once (GAE-YOLO) algorithm. The lightweight architecture of the GAE-YOLO framework is achieved through the replacement of standard convolutional layers with Ghost Convolution (GhostConv) modules, while detection accuracy is significantly improved by the integration of both AReLU activation functions and Effective Intersection over Union (E-IoU) loss optimization. The system, implemented on a Jetson TX2 embedded platform, also incorporates ZED stereo vision for 3D localization and a PyQt6-based visualization platform.

**Results:**

When implemented on Jetson TX2, the system achieving 93.5% mean Average Precision at 50% intersection over union (mAP@50) at 10.2 frames per second (FPS), which can be optimized to 27 FPS by employing TensorRT acceleration and 720p resolution for scenarios demanding higher throughput. Furthermore, it establishes standardized assessment systems for tomato maturity and yield prediction, and offers integrated modules for disease diagnosis and agricultural large language model consultation.

**Discussion:**

This work establishes a new paradigm for edge computing in agriculture while providing critical technical support for smart farming development.

## 1 Introduction

Tomato (Solanum lycopersicum), as a globally significant economic crop rich in essential vitamins, plays a pivotal role in agricultural production and human nutrition. In recent years, the global cultivation area of tomato has experienced substantial expansion, driven by increasing market demand. However, industrialized tomato production continues to face critical technical constraints, including inefficient harvesting systems, inconsistent fruit maturity, and challenges in accurate disease identification, which collectively hinder sustainable development of the industry (Liu J. et al., 2020). Three major challenges are identified in conventional tomato production: (1) Inefficient harvesting processes: Tomato yield and economic returns are significantly impacted by harvesting efficiency. Current operations remain heavily dependent on manual labor due to the complexity of maturity assessment and limited mechanization, resulting in elevated labor costs and suboptimal productivity. (2) Imprecise pest/disease management: During ripening stages, tomato crops are particularly vulnerable to pest infestations and diseases, often leading to substantial yield reduction. Traditional monitoring approaches relying on manual field inspections and empirical judgments are characterized by delayed responses and high misidentification rates. Under large-scale cultivation conditions, such labor-intensive methods fail to provide comprehensive coverage, creating significant prevention gaps. (3) Scientific cultivation limitations: The expansion of cultivation areas has exacerbated management challenges, particularly in regions with limited agricultural expertise. Improper pesticide application not only compromises disease control efficacy but also risks secondary contamination through excessive residues. Furthermore, the prevalent overuse of broad-spectrum pesticides in technician-deficient regions increases production costs while aggravating environmental pollution risks.

Recent advancements in artificial intelligence (AI) have provided transformative technological support for intelligent agricultural systems. Particularly in tomato production, AI-based computer vision and deep learning technologies have emerged as innovative solutions to conventional harvesting challenges. Studies have demonstrated that agricultural robotic systems equipped with high-precision visual recognition modules are capable of real-time morphological characterization, enabling precise robotic harvesting operations through mechanical arm guidance. The evolution of fruit detection methodologies has witnessed a paradigm shift from traditional approaches to deep learning techniques. Conventional detection methods, including single-feature analysis, multi-feature fusion, and threshold segmentation algorithms, have been shown to exhibit significant limitations when applied to complex tomato growth patterns characterized by fruit occlusion and foliar obstruction. In contrast, deep learning approaches have demonstrated substantial improvements in both recognition accuracy and environmental robustness through autonomous multi-scale feature extraction. This technological advancement provides fundamental support for developing intelligent harvesting systems that address the critical limitations of traditional methods, particularly their inefficiency and labor-intensive nature. Afonso et al. applied MaskRCNN for tomato detection in greenhouse images, by leveraging deep learning to handle variability, implicitly learn depth, and enable accurate fruit counting and background elimination in real-world conditions (Afonso et al., 2020). Liu et al. proposed YOLO-Tomato, an improved YOLOv3 model with dense architecture and circular bounding boxes, enhancing tomato detection accuracy under challenging conditions like occlusion and overlap, outperforming state-of-the-art methods (Liu G. et al., 2020). Liu et al. proposed SM-YOLOv5, a lightweight model based on YOLOv5 and MobileNetV3, achieving 98.8% mAP for small-target tomato detection in plant factories, meeting real-time requirements for picking robots with reduced computational cost (Wang et al., 2023). Wang et al. proposed an improved Faster R-CNN with CBAM and FPN for tomato young fruit detection, achieving 98.46% mAP and 0.084s/image, addressing color similarity, occlusion, and overlap challenges for real-time precision (Wang et al., 2021). Zhou et al. proposed a real-time tomato maturity detection method using YOLOv4 for fruit detection and RGB color analysis with K-means clustering for maturity estimation in greenhouse environments (Zhou et al., 2021). Chen et al. enhanced YOLOv3 for cherry tomato detection by integrating a dual-path network for richer small-target features, multi-scale prediction, and improved K-means++ clustering for anchor box optimization (Chen et al., 2021). Su et al. proposed SE-YOLOv3-MobileNetV1, integrating depthwise separable convolution, Mosaic augmentation, K-means clustering, and SE attention for efficient tomato maturity classification in greenhouse environments, optimized for embedded systems (Su et al., 2022). Rong et al. developed YOLOv5-4D, combining RGB-depth fusion and ByteTrack for tomato cluster tracking, with a specific counting region method to enhance stability and accuracy in greenhouse yield estimation (Rong et al., 2023). Tian et al. proposed TF-YOLOv5s, enhancing YOLOv5s with C3Faster, depth-wise separable convolution, EIoU loss, and SE modules for efficient tomato flower and fruit detection in natural environments, optimized for edge computing deployment (Tian et al., 2024).

Recent studies have further advanced lightweight architecture design. Wang et al. introduced a novel combination of switchable atrous convolution for dynamic receptive field adjustment and wavelet transform convolution for multi-frequency feature decomposition, which effectively preserved critical edge details of occluded tomatoes in greenhouse environments (Wang et al., 2025). Concurrently, Zhang et al. addressed the challenge of detecting dense, small-sized disease spots by integrating a Normalized Wasserstein Distance loss that stabilized the learning process for tiny features, along with a lightweight hybrid attention mechanism to enhance focus on discriminative regions (Zhang and Jiang, 2025). Hao et al. achieved a significant reduction in parameters and computational load by designing a GSim module and replacing standard components with C3Ghost and BiFPN structures, while maintaining high ripeness detection accuracy in complex environments (Hao et al., 2025). Furthermore, Deng et al. implemented a fundamentally different approach through embedding Sobel operators directly into the network stem for explicit edge feature extraction from the initial stage, combined with a weighted Focaler-IoU loss that achieved state-of-the-art accuracy while demonstrating practical deployment capability on edge hardware platforms (Deng et al., 2025). However, these models remain isolated perception modules, creating a perception-action gap due to the lack of integrated 3D coordination for robotics. The present study bridges this gap through the GAE-YOLO system, which couples detection with ZED stereo vision to form a complete detection-localization-analysis-decision pipeline. This integration enables precise spatial localization for robotic manipulation alongside maturity assessment and yield estimation, establishing a new paradigm for comprehensive edge intelligence in agriculture.

The early detection of plant diseases has been recognized as a critical factor in maintaining agricultural productivity. Initial disease manifestations on plant foliage are frequently associated with significant reductions in both crop yield and quality. Conventional diagnostic approaches, which predominantly rely on visual inspection by human experts, have been demonstrated to suffer from two fundamental limitations: operational inefficiency and substantial subjective bias. Recent advances in artificial intelligence have led to the development of deep learning-based image recognition systems. These systems have shown considerable potential in plant pathology applications due to their exceptional capabilities in automated feature extraction and diagnostic accuracy. Mokhtar et al. proposed an image processing approach using GLCM for texture analysis and SVM with linear kernel for classifying healthy and infected tomato leaves, achieving high accuracy with N-fold cross-validation (Mokhtar et al., 2015b). Mokhtar et al. proposed a method using Gabor wavelet transform for feature extraction and SVMs with alternate kernel functions to detect and classify tomato leaf diseases, achieving high accuracy and reliability (Mokhtar et al., 2015a). Fuentes et al. analyzed non-destructive image-based techniques for detecting tomato plant diseases, emphasizing the importance of accurate data collection to reduce agricultural production and economic losses (Fuentes et al., 2016). Mohanty et al. developed a deep convolutional neural network (CNN) for crop disease diagnosis using a large public image dataset, demonstrating the potential for smartphone-assisted global disease identification (Mohanty et al., 2016). TM et al. proposed a modified LeNet CNN for tomato leaf disease detection, utilizing minimal computing resources and automatic feature extraction to achieve efficient and accurate classification under challenging conditions (Tm et al., 2018). Ji et al. proposed a lightweight YOLOv8-based method for tomato leaf disease detection, integrating enhanced IoU, AKConv, and GSConv to improve localization accuracy and reduce computational complexity for efficient disease recognition (Ji et al., 2024). Phan et al. proposed four deep learning frameworks combining Yolov5m with ResNet50, ResNet-101, and EfficientNet-B0 for classifying tomato fruit into ripe, immature, and damaged categories, demonstrating potential for automated harvesting (Phan et al., 2023). Umar et al. proposed an improved YOLOv7 model with SimAM, DAiAM, and MPConv for accurate tomato leaf disease detection, combined with SIFT-based segmentation and CNN classification for enhanced feature extraction and disease identification (Umar et al., 2024). This non-destructive intelligent detection approach has been demonstrated to significantly improve diagnostic efficiency while providing crucial technical support for timely and effective disease management, thereby playing a pivotal role in ensuring tomato yield and quality.

Agricultural large language models (LLMs), as an important artificial intelligence technology, have demonstrated significant potential in various agricultural applications, particularly in crop identification and disease early warning systems. Wang et al. introduced Agri-LLaVA, a knowledge-infused multimodal conversation system for agriculture, leveraging a novel 400,000-entry dataset covering 221 pests and diseases. The system enhances visual understanding and pest control, with open-source resources to advance agricultural LMM research (Wang et al., 2024). Yu et al. proposed AgriVLM, a framework fine-tuning visual language models with Q-former and Low-Rank adaptation for cross-modal fusion of agricultural data, enhancing crop disease and growth stage recognition through multimodal analysis (Yu and Lin, 2024).Wang et al. developed an intelligent agricultural Q&A system using LLMs, fine-tuned with Lora and Prompt-tuning for named entity recognition and question answering, enhancing agricultural knowledge dissemination for rural revitalization (Wang et al., 2023). Zhang et al. proposed IPM-AgriGPT, a Chinese LLM for pest management, using a G-EA framework, ACR-CoTD, and LoRA techniques to optimize dynamic reasoning and reduce reliance on labeled data for agricultural intelligence (Zhang et al., 2025). Large model technology has demonstrated outstanding performance in agricultural multimodal dialogue and visual comprehension, providing novel insights and methodologies for addressing agricultural disease challenges.

As evidenced by current research, computer vision-based intelligent tomato detection has emerged as a critical component in automated harvesting systems, where detection accuracy directly determines the operational efficiency of subsequent robotic processes. However, existing approaches are constrained by two fundamental limitations: On the one hand, the practical deployment of high-complexity models in agricultural environments presents significant challenges, on the other hand most current developments remain confined to theoretical algorithm research without comprehensive implementable solutions. To identify the most suitable lightweight architecture for agricultural edge detection, we systematically evaluated mainstream backbones and identified GhostConv as offering a superior trade-off between accuracy and efficiency, which subsequently formed the core of our GAE-YOLO model. To address these practical challenges in automated tomato harvesting, an intelligent computer vision-based tomato detection and management system is proposed in this study. This system aims to resolve core technical barriers in agricultural automation through systematic integration of advanced visual algorithms with edge computing technologies, thereby achieving a crucial transition from theoretical research to practical application. The primary contributions of this work are manifested in four key aspects:

1. Algorithm Optimization: The conventional convolutional structure was replaced with Ghost-conv modules, achieving a significant reduction in model parameters. The E-IoU loss function and AReLU activation function were implemented to enhance detection accuracy, enabling high-precision identification of tomato fruits and foliar diseases.
2. System Deployment: A novel three-dimensional tomato detection framework was developed by integrating ZED binocular vision with the Jetson TX2 edge computing platform. This integrated system facilitates real-time dynamic analysis and provides precise guidance for robotic harvesting operations.
3. Scientific Management: Standardized algorithms for tomato maturity evaluation and single-plant yield estimation were established, providing quantitative data support for precision agriculture practices.
4. Application Functionality: A cross-platform visualization interface was developed with modular design, incorporating multidimensional diagnostic reporting and optimized agricultural language models to deliver intelligent disease prevention and control solutions.

Experimental results demonstrate that the proposed system significantly outperforms conventional approaches in multiple critical aspects, including tomato detection accuracy, disease identification precision, maturity assessment reliability, and prevention strategy effectiveness. This study has established not only a practical and implementable solution for agricultural automation, but also a scalable technical framework that can be readily adapted for intelligent management of various cash crops. The proposed system exhibits substantial academic significance while demonstrating considerable potential for industrial deployment.

## 2 Materials and methods

### 2.1 Construction of data set

#### 2.1.1 Tomato detection datasets construction

**Figure 1:**
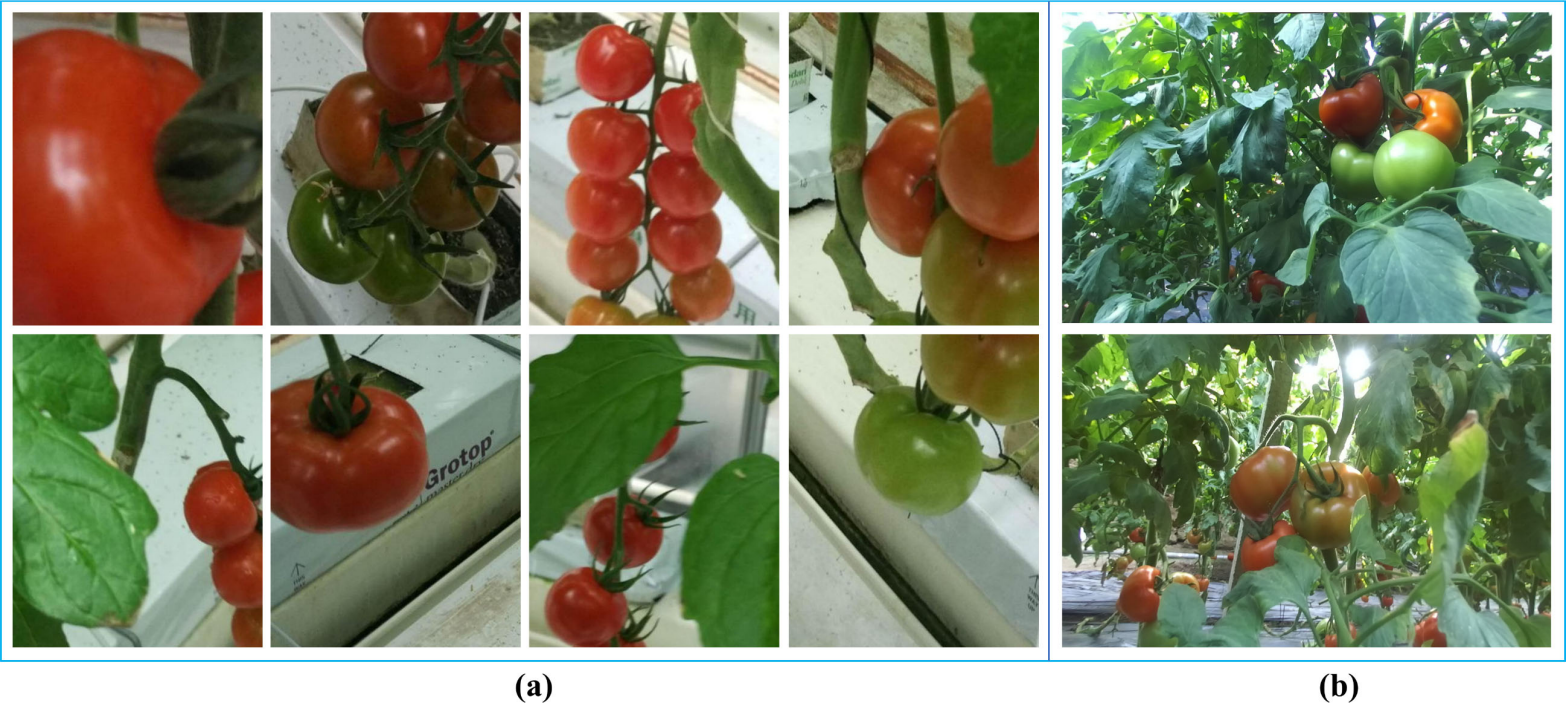
Partial tomato datasets. **(a)** Example images from the tomato dataset. **(b)** Tomato images captured by a ZED stereo camera.

**Figure 2:**
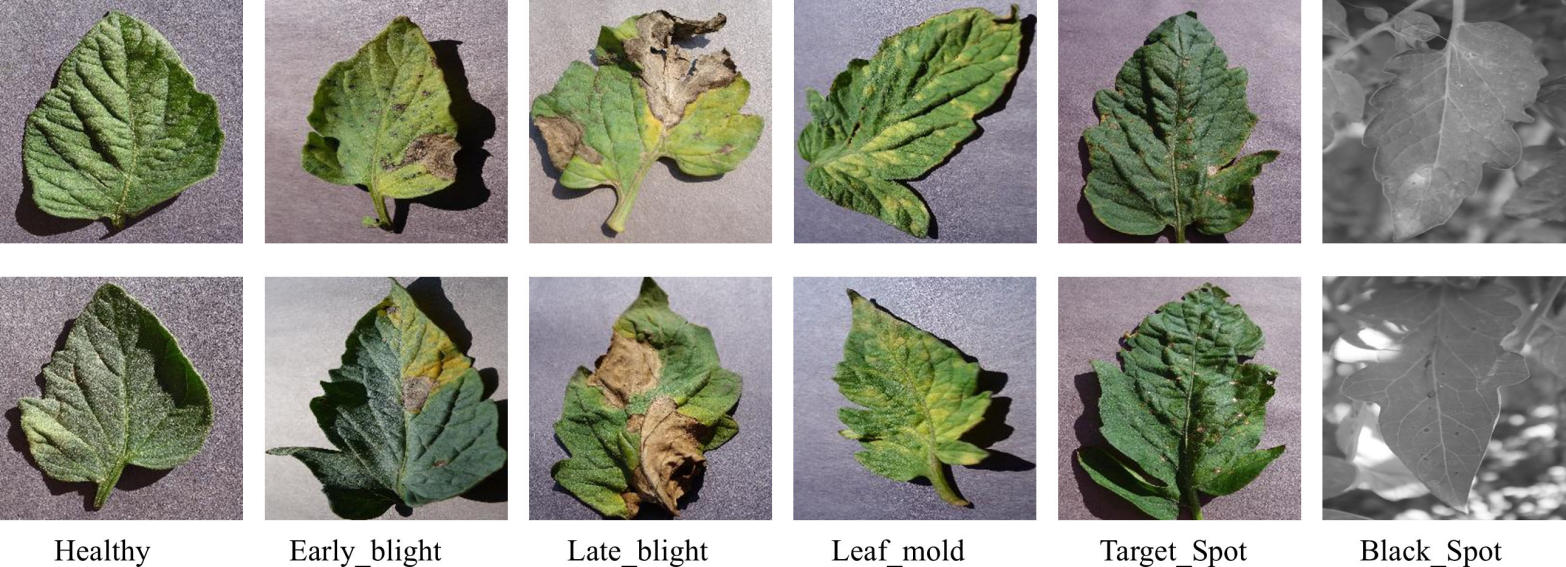
Partial foliar disease datasets.

Tomato detection datasets construction: A multi-source data fusion strategy was employed to construct a comprehensive tomato detection datasets. The datasets comprises two primary components: (1) a standardized benchmark datasets containing 895 high-resolution tomato images obtained from Kaggle, with each image professionally annotated in PASCAL VOC format (**Figure 1a**), and (2) supplementary field-collected data acquired from greenhouse environments in Shouguang City, Shandong Province (36°51’19.73”N, 118°47’26.35”E) using ZED binocular cameras. The field collection protocol ensured diverse samples by capturing tomato plants at various growth stages under multiple lighting conditions and viewing angles (**Figure 1b**).

#### 2.1.2 Foliar disease detection datasets

The foliar disease datasets used in this study was obtained from the Kaggle platform, comprising over 700 tomato leaf images collected from both laboratory and field environments. The dataset contains six disease categories and one healthy class, including common tomato pathogens: Bacterial spot, Early blight, Late blight, Leaf mold, Target Spot, and Black Spot. The data has been augmented of using multiple advanced techniques such as image flipping, Gamma correction, noise injection, PCA color augmentation, rotation, and scaling. Some foliar disease datasets are shown in **Figure 2**.

#### 2.1.3 Image acquisition and processing facilities

**Figure 3:**
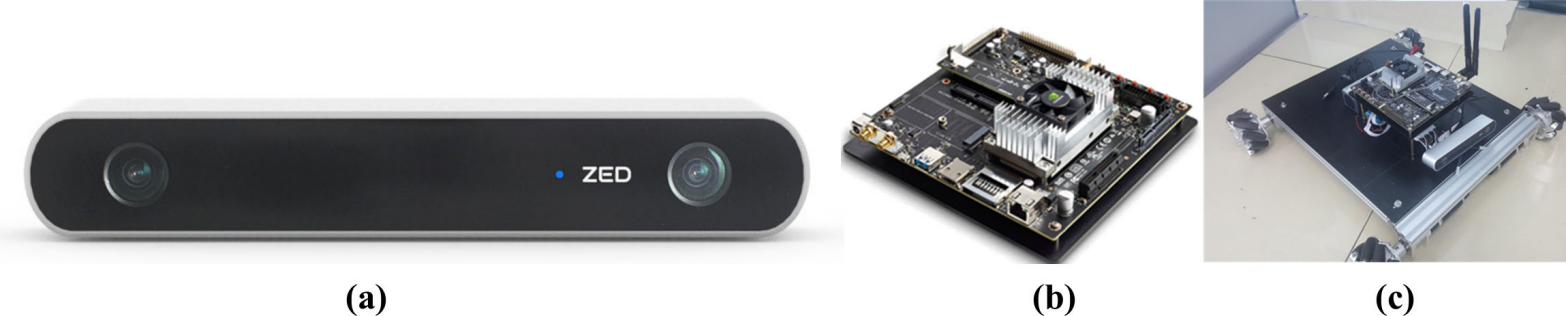
Hardware facilities for image acquisition and processing. **(a)** ZED binocular vision camera. **(b)** Jetson TX2 edge computing module. **(c)** McNam wheel omnidirectional mobile platform.

This study focuses on intelligent tomato recognition and detection in real-world agricultural settings, moving beyond traditional static datasets-based algorithm research. To address the complex imaging challenges in greenhouse environments, a mobile image acquisition system was developed, comprising three core components: (1) a ZED binocular vision camera (**Figure 3a**), which provides high-precision depth perception and 4K resolution imaging; (2) a McNam wheel omnidirectional mobile platform (**Figure 3c**), enabling flexible and adaptive data collection; and (3) a Jetson TX2 edge computing module (**Figure 3b**), responsible for real-time image processing and analysis. This integrated system effectively overcomes the challenges posed by variable lighting conditions and foliar obstructions in greenhouse environments, ensuring reliable data acquisition for subsequent tomato detection and maturity analysis.

### 2.2 Proposed methods

**Figure 4:**
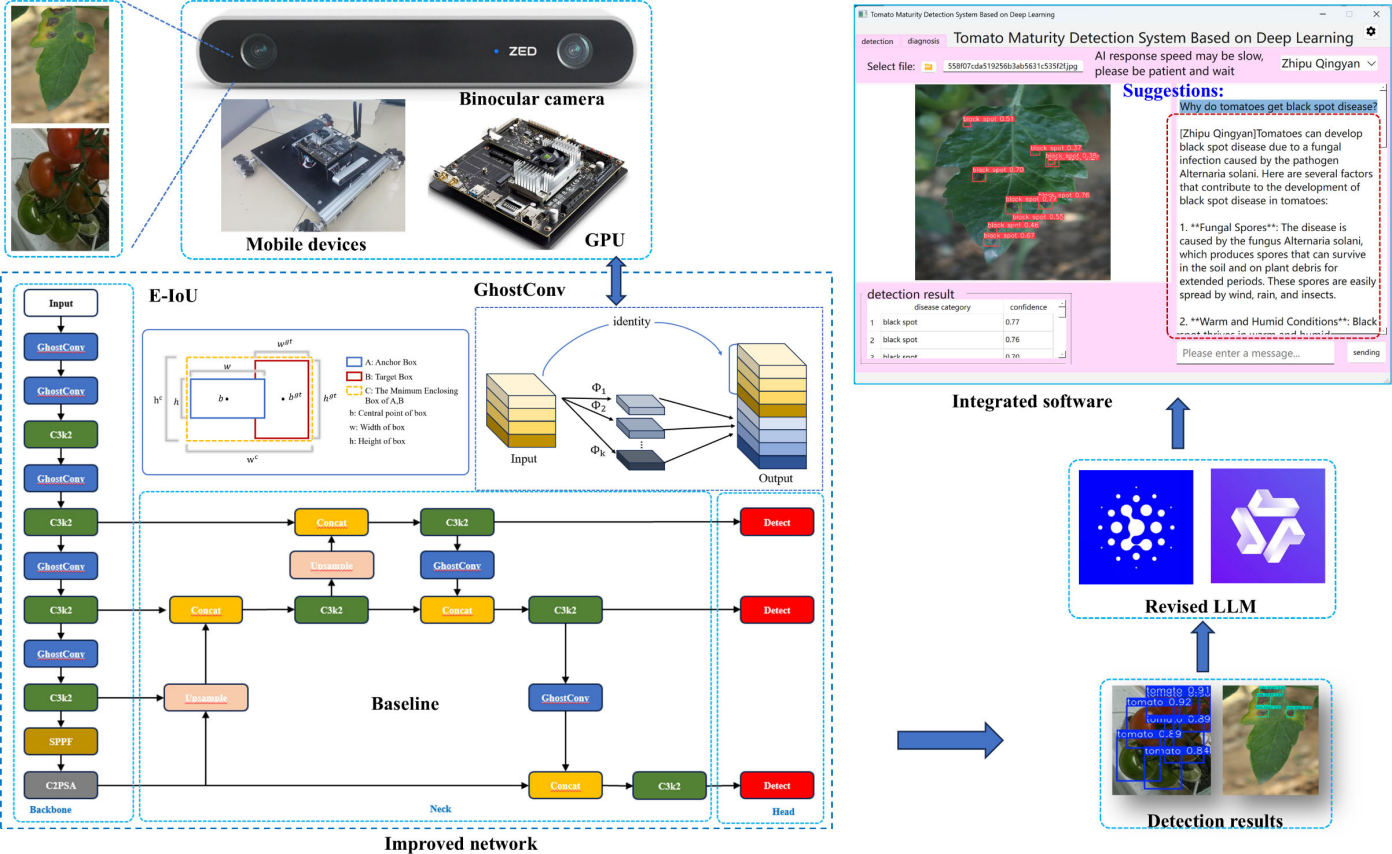
Technical roadmap of tomato smart management system.

This study presents an intelligent tomato detection and management system based on the GAE-YOLO model, which achieves end-to-end process optimization—from fruit detection to disease diagnosis and ripeness assessment—through the integration of multiple advanced technologies. Methodologically, the system is built upon four core technical modules: (1) a lightweight GAE-YOLO object detection framework for high-precision identification of tomato fruits and disease symptoms; (2) a three-dimensional vision system integrated with a ZED depth camera to establish accurate spatial coordinate calculations for robotic harvesting; (3) a multi-scale feature fusion algorithm designed for tomato ripeness classification and yield estimation; and (4) visualization software that combines disease diagnosis with LLMs interaction, enabling a modular “detection-diagnosis-decision” closed-loop management system. The collaborative workflow of these modules is demonstrated in the technical roadmap (**Figure 4**).

#### 2.2.1 Lightweight GAE-YOLO

**Figure 5:**
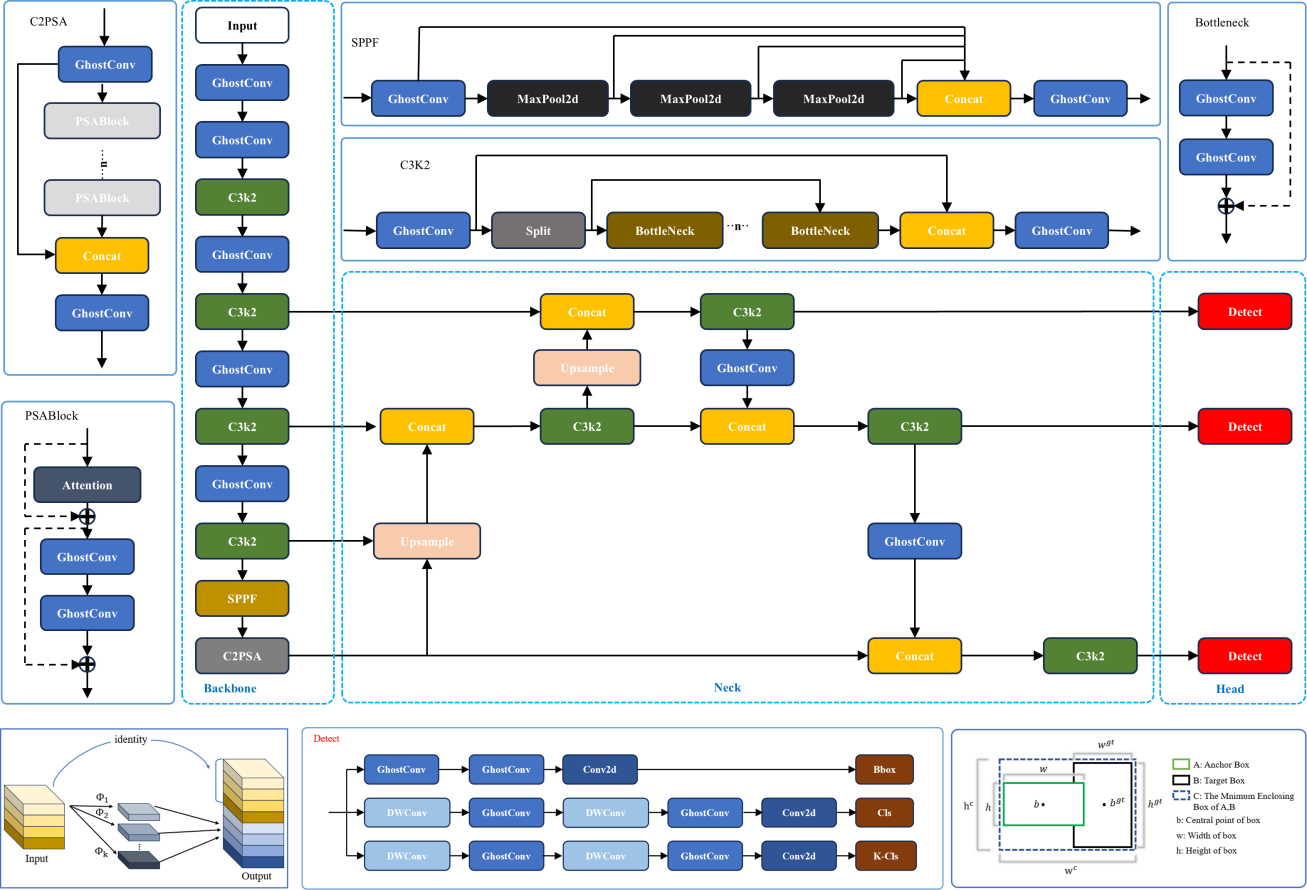
GAE-YOLO network architecture diagram.

This study proposes an innovative lightweight object detection model, GAE-YOLO, which systematically optimizes the Ultralytics YOLOv11-nano (YOLOv11n) architecture. As shown in **Figure 5**, GAE-YOLO achieves significant improvements in balancing model efficiency and detection accuracy through three key technological innovations: (1) Lightweight architecture design: GhostConv modules are employed to replace conventional convolution operations, reducing parameters while maintaining feature extraction capability through a feature map redundancy utilization mechanism, thereby improving inference speed. (2) Adaptive feature enhancement: Parametric AReLU activation functions are introduced to replace standard SiLU, dynamically adjusting negative interval slopes to better capture key morphological features of tomato fruits. (3) Optimized training strategy: The E-IoU loss function is adopted, integrating direction-aware terms and scale-sensitive factors to effectively address recognition deviations in tomato occlusion scenarios. These innovations enable GAE-YOLO to maintain detection accuracy while achieving lightweight deployment on mobile devices (Jetson TX2) for real-time tomato detection.

##### 2.2.1.1 Lightweight GhostConv design

**Figure 6:**
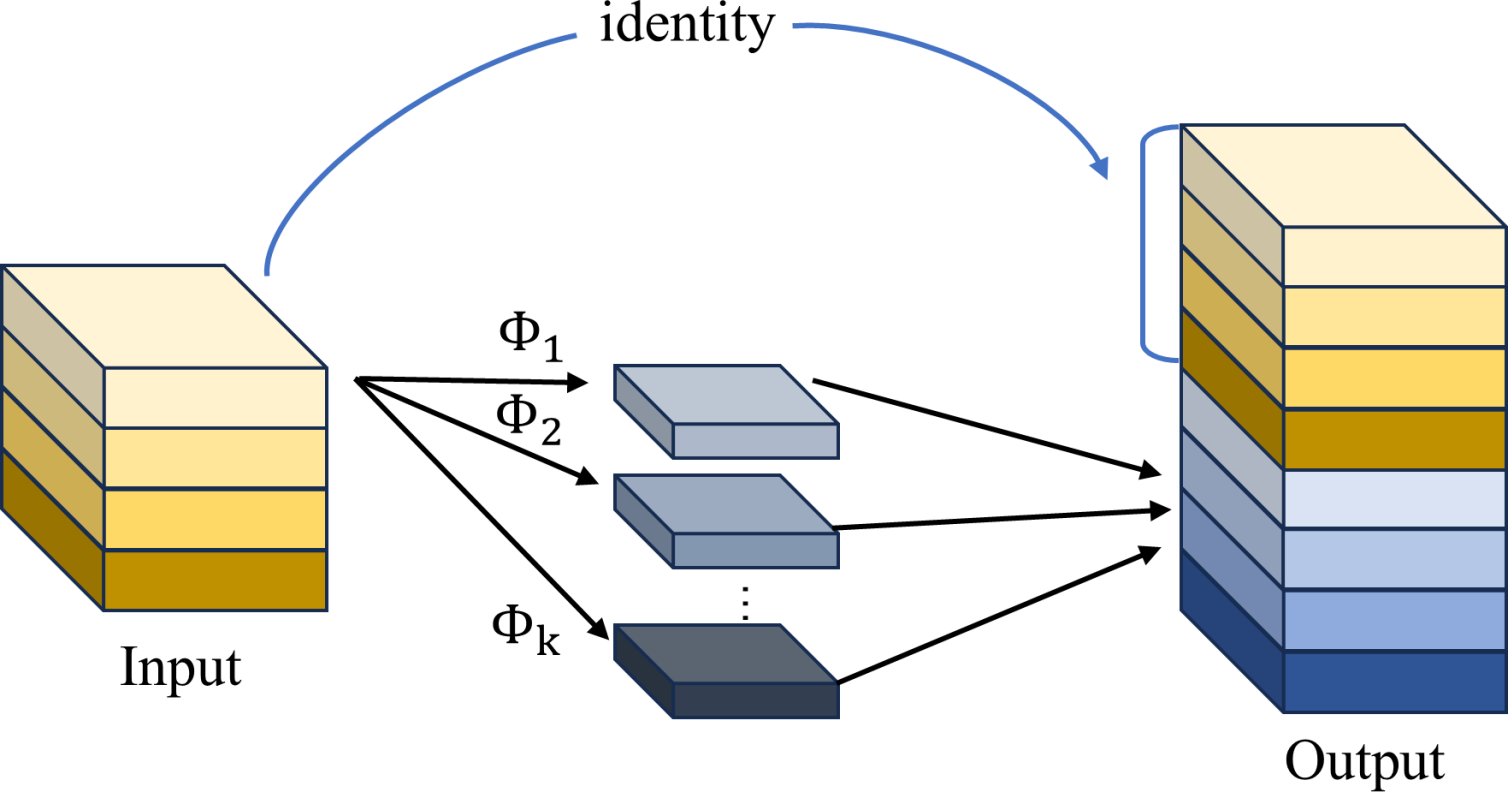
The GhostConv module.

CNNs have demonstrated remarkable success in computer vision applications; however, their deployment on resource-constrained devices remains challenging. Traditional CNN architectures rely on stacked convolutional layers for feature extraction, which, while achieving high detection accuracy, incur substantial computational costs. To address this limitation, this study introduces GhostConv as an innovative replacement for conventional convolution operations. As illustrated in **Figure 6**, the GhostConv module employs depth-wise convolution as its foundational operation, decoupling channel dependencies by performing independent convolution operations for each feature channel. This design significantly reduces computational complexity while maintaining representational capacity. The module further enhances efficiency through a secondary linear transformation layer, which generates supplementary ghost feature maps at minimal computational overhead. Collectively, these mechanisms enable efficient yet powerful feature extraction.

The GhostConv operation proceeds as follows: A minimal set of conventional convolution kernels are first applied to the input feature map X to extract intrinsic feature maps $Y^{\prime }$, where X denotes the input features and $f^{\prime }$ represents the number of convolutional layers, as shown in Equation 1.

(1) $$Y^{\prime }=X\;*\;f^{\prime }$$

A linear transformation is then applied to the output intrinsic feature maps $Y^{\prime }$ to generate ghost feature maps (Equations 2, 3).

(2) $$y_{ij}={\text{Φ}}_{i,j}(y_{i}^{'}),\;\;\forall i=1,\ldots ,m,\;\;j=1,\ldots ,s$$

(3) $$Y=[y_{11},y_{12},\ldots ,y_{1s},\ldots ,y_{ms}]$$

The intrinsic feature maps obtained in the first step and the ghost feature maps generated in the second step are concatenated to produce the final output feature maps.

The innovation of this design is demonstrated through optimized feature learning mechanisms that significantly reduce the model’s computational cost for non-critical features in tomatoes and their foliage. Specifically, conventional convolution operations are replaced by a lightweight kernel combination strategy integrated with efficient linear transformations. This approach maintains model performance stability while substantially decreasing computational resource requirements, making it particularly suitable for efficient deployment on resource-constrained edge devices such as the Jetson TX2 platform.

##### 2.2.1.2 Improving activation functions with AReLU

Activation functions, serving as fundamental components of deep neural networks, critically influence model performance through their impact on both representational capacity and learning dynamics. While traditional static activation functions (e.g., ReLU, ELU) demonstrate computational efficiency, they are constrained by two inherent limitations. First, fixed nonlinear transformation patterns that fail to adapt to hierarchical feature distributions. Second, uniform activation thresholds that lack adaptive feature selection capabilities. Although existing parametric activation functions (e.g., PReLU, SReLU) partially mitigate these issues through learnable parameters, their feature selection mechanisms remain suboptimal in precision. To address these challenges, this study proposes an innovative framework that deeply integrates attention mechanisms with activation functions, wherein the original SiLU is replaced by AReLU (Chen et al., 2020). The used AReLU achieves fine-grained feature regulation through three core mechanisms: (1) a dynamic threshold mechanism that automatically adjusts activation thresholds based on feature importance. (2) a context-aware module that modulates activation intensity using local feature statistics. and (3) a multi-scale fusion technique that enables layer-specific activation pattern learning.

**Figure 7:**
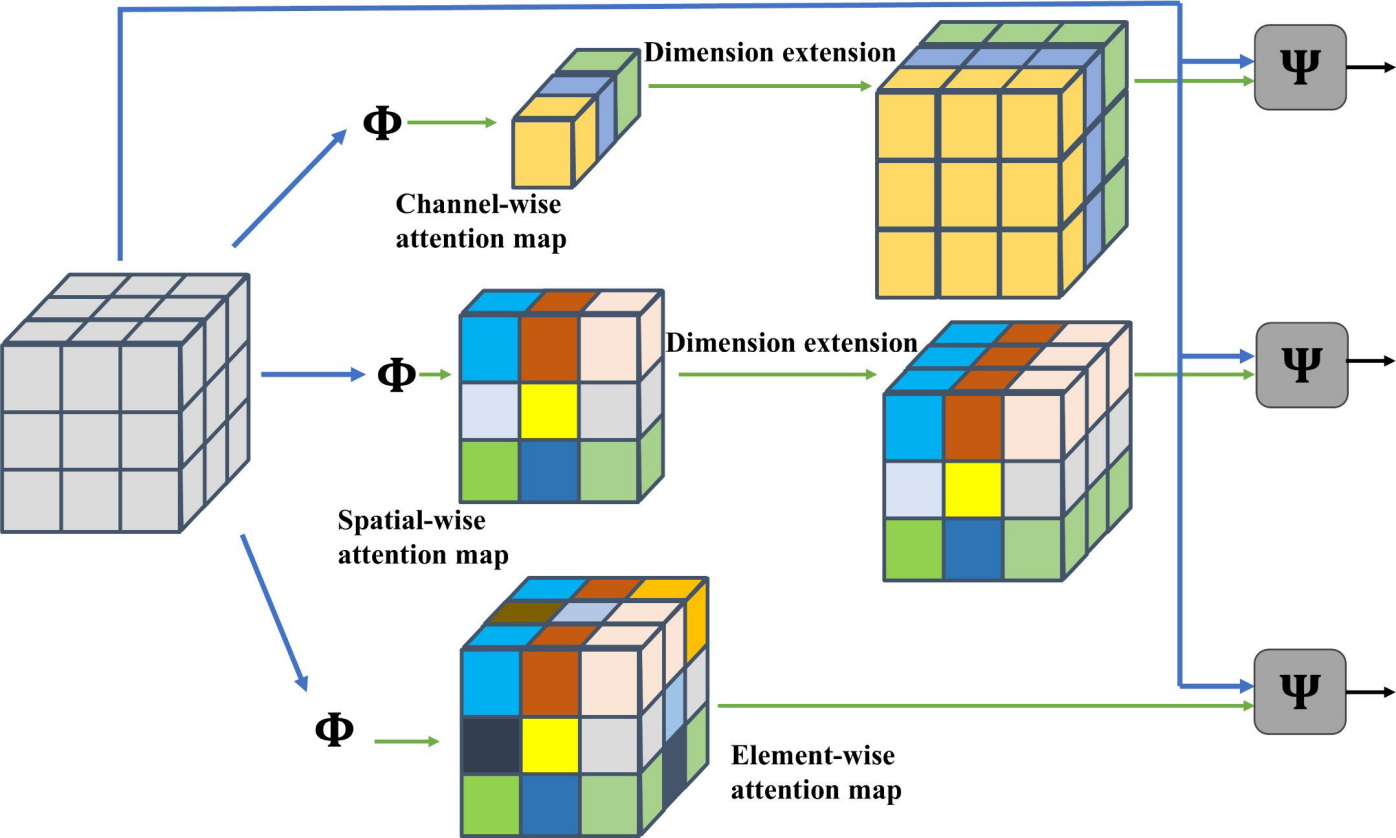
Schematic diagrams of different attention mechanisms.

**Figure 7** presents schematic diagrams of various attention mechanisms, including: Channel-wise Attention Mechanism, Spatial-wise Attention Mechanism, and Element-wise Attention Mechanism.

This study employs an Element-wise Learned Sign Attention (ELSA) module to learn sign-dependent attention weights for pre-activation feature maps, where distinct learnable parameters are assigned to positive and negative elements respectively, enabling dynamic regulation through amplifying positive elements while suppressing negative ones. Considering a feature volume $V=\{v_{i}\}\in R^{W\cdot H\cdot C}$, the work compute an element-wise attention map $S=\{s_{i}\}\in R^{W\cdot H\cdot C}$,where ϕ can be implemented via a neural network, as shown in Equation 4.

(4) $$s_{i}=\text{Φ}(v_{i},\text{Θ})=\{\begin{array}{c} C(\alpha ),\;\;v_{i}<0\\ \sigma (\beta ),\;\;v_{i}\geq 0 \end{array}$$

For a feature element $v_{i}$, its attention value $s_{i}$ is governed by both negative and positive elements, with the positive element weight being computed via the sigmoid function, where $\text{Θ}=\{\text{α},\text{β}\}\in R^{2}$ is learnable parameters. C(·) clamps the input variable into [0.01, 0.99]. σ is the sigmoid function. The attention weights are combined with input features through element-wise multiplication. The modulation function Ψ is defined as Equation 5.

(5) $$u_{i}=\text{Ψ}(v_{i},s_{i})=v_{i}\cdot s_{i}$$

This study implements function Φ in ELSA using a neural layer with learnable parameters αand β: AReLU is constructed by combining the ELSA module with conventional ReLU, thereby yielding a learnable activation function, as shown in Equation 6.

(6) $$\text{F}(x_{i},\text{α},\text{β})=\{\begin{array}{c} C(\alpha )x_{i},\;\;x_{i}<0\\ (1+\sigma (\beta ))x_{i},\;\;x_{i}\geq 0 \end{array}$$

where $X=\{x_{i}\}$ represents the input to the current layer. Here, ReLU provides fundamental nonlinear activation, while ELSA learns residual weights to further amplify positive elements and moderately suppress negative ones.

This innovative design not only preserves the computational efficiency of traditional ReLU but also incorporates feature selection adaptability through attention mechanisms, significantly improving the model’s capability to learn multi-scale features in tomato vision tasks. Specifically, AReLU employs a learnable parameterization mechanism that dynamically adjusts activation patterns across different feature map levels. This adaptation enables the network to effectively capture multi-scale fruit morphological features, ranging from microscopic to macroscopic details, while providing enhanced nonlinear expressiveness for precise tomato detection and foliar disease identification.

##### 2.2.1.3 Optimized Focal-EIoU loss function

**Figure 8:**
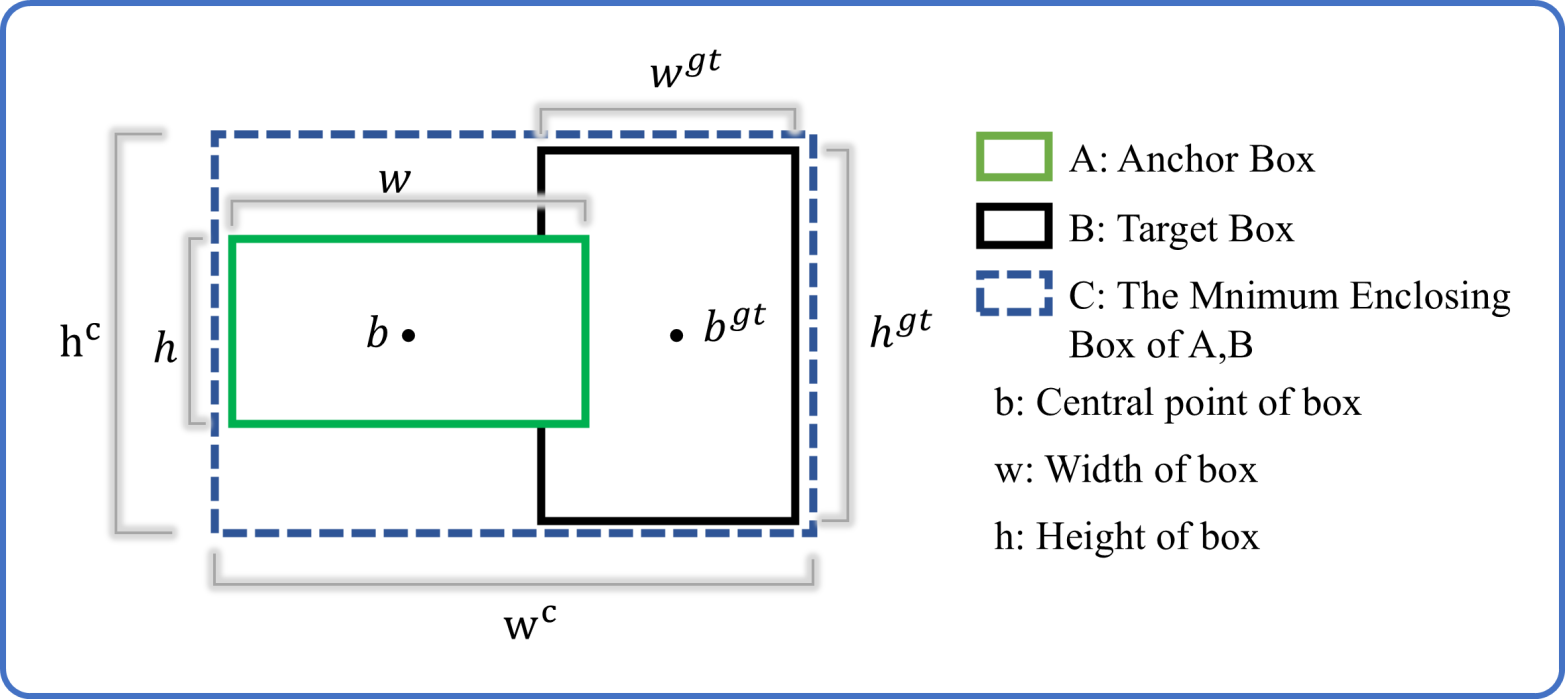
Schematic diagram of EIOU bounding box regression.

This study presents a comprehensive analysis of the bounding box regression optimization challenge in the YOLOv11 object detection framework. While the conventional CIoU loss function demonstrates improved bounding box prediction accuracy, two critical limitations are identified: (1) The coupled aspect ratio penalty term fails to precisely characterize the dimensional discrepancies between target and anchor boxes in width and height; and (2) The scale sensitivity in bounding box regression is inadequately addressed, leading to suboptimal convergence rates during model optimization and constrained localization precision. These limitations become particularly pronounced in tomato detection scenarios, especially when processing densely clustered or partially occluded targets, where coordinate localization accuracy is substantially compromised. To address these issues, the Focal-EIoU loss function is implemented in this study, with its diagram illustrated in **Figure 8**.

The EIoU penalty term builds upon the CIoU framework by decoupling the aspect ratio influence factor and separately computing width and height discrepancies between target and anchor boxes. This loss function comprises three components: overlap loss, center distance loss, and width-height loss, with the first two components maintaining the CIoU methodology. This design not only preserves the beneficial properties of CIoU loss but also directly minimizes width and height deviations between target and anchor boxes through EIoU, resulting in accelerated convergence and enhanced localization performance. The penalty term is formulated as shown in Equation 7.

(7) $$L_{EIoU}=L_{IoU}+L_{dis}+L_{asp}=1-IoU+\frac{\rho^{2}(b,b^{gt})}{{\left(w^{c}\right)}^{2}+{\left(h^{c}\right)}^{2}}+\frac{\rho^{2}(w,w^{gt})}{{\left(w^{c}\right)}^{2}}+\frac{\rho^{2}(h,h^{gt})}{{\left(h^{c}\right)}^{2}}$$

where $w^{c}$ and $h^{c}$ are the width and height of the smallest enclosing box covering the two boxes. Addressing the inherent imbalance in bounding box regression training samples, where high-quality anchor boxes are significantly outnumbered by low-quality samples, requires special consideration, as poor-quality samples can generate excessively large gradients that adversely affect the training process. However, conventional reweighting methods cannot be directly applied to IoU-based losses. To mitigate this issue, the Focal-EIoU loss is applied (Zhang et al., 2022), which leverages the IoU value to reweight the EIoU loss. This design places greater emphasis on difficult-to-classify samples during optimization, thereby improving the overall performance of the target detection algorithm. The formulation is given in Equation 8.

(8) $$L_{Focal-EIoU}=IoU^{\gamma }L_{EIoU}$$

The parameter γ controls the suppression intensity for outlier samples. As derived from the formulation, higher IoU values correspond to increased loss magnitudes, effectively implementing a weighting mechanism that assigns greater loss values to superior regression targets - thereby enhancing regression accuracy. The γvalue directly determines the model’s focus level on challenging samples. In tomato detection tasks, this mechanism exhibits two key advantages: First, by allocating higher loss weights to high-IoU samples, the positioning accuracy for mature tomatoes is significantly improved. Second, this dynamic weighting strategy effectively balances the contributions of varying-quality samples to model optimization, enabling the detection algorithm to achieve enhanced robustness in complex agricultural environments.

#### 2.2.2 Tomato maturity calculation based on GAE-YOLO

Tomato maturity detection and yield estimation play a pivotal role in precision agriculture by providing critical decision-making support. This study leverages the GAE-YOLO detection algorithm combined with ZED binocular depth cameras to accomplish three core functionalities: (1) accurate maturity stage classification (unripe/ripe/overripe) through multi-feature analysis; (2) precise calculation of three-dimensional spatial coordinates and size parameters using depth vision technology; and (3) intelligent yield prediction at both plant and area-unit levels by integrating tomato variety characteristics with evaluation algorithms. Furthermore, real-time monitoring of tomato maturity distribution enables optimal harvesting time determination. A tomato maturity assessment model was developed based on the GAE-YOLO framework, incorporating a multi-feature weighted fusion strategy. In this model, three key feature sets were identified as maturity indicators: (1) HSV color space characteristics, where hue serves as the most visually discriminative maturity parameter; (2) size characteristics, which exhibit high inter-variety differentiation when combined with cultivar-specific traits; and (3) shape characteristics, which primarily assist in overripe fruit identification while showing limited discrimination between unripe and ripe stages. The relative importance of these maturity indicators is quantitatively presented in **Table 1**.

**Table 1:** Key characteristics for judging tomato maturity.

Feature	Unripe tomato	Ripe tomato	Overripe tomato
Color	Green or light red	Uniform bright red	Dark red with local blackening
Size	Smaller	Variety-standard size	Shriveled/swollen
Shape	Regular round	Regular round	Deformed/sunken/shriveled

##### 2.2.2.1 Tomato maturity judgment based on color space

In this study, the HSV color space was selected as the primary metric for tomato maturity assessment. Color distributions within GAE-YOLO detection bounding boxes were systematically analyzed through targeted extraction. Compared to RGB color space, HSV demonstrates superior suitability for tomato ripeness evaluation due to three key advantages: (1) The hue (H) channel exhibits strong correlation with ripeness progression, enabling direct assessment through H-values without requiring complex threshold combinations as in RGB; (2) Enhanced robustness against lighting variations, making it particularly suitable for greenhouse environments with fluctuating illumination; and (3) Independent V-channel (value) that maintains color interpretation stability across lighting conditions while remaining decoupled from H and S channels.

It should be emphasized that in **Table 2**, the H (Hue) parameter represents color types through angular measurements $\left(0^{{}^{\circ}},360^{{}^{\circ}}\right)$. This angular representation exhibits two unique characteristics: (1) The $\left(0^{{}^{\circ}},360^{{}^{\circ}}\right)$ range is not mathematically continuous due to its periodic nature (where 0° and 360° represent identical colors), and (2) Distinct maturity stages correspond to specific hue ranges - unripe tomatoes display green/yellow hues $\left[50^{{}^{\circ}},100^{{}^{\circ}}\right)$, ripe tomatoes exhibit red hues $\left[0^{{}^{\circ}},20^{{}^{\circ}})\cup [330^{{}^{\circ}},360^{{}^{\circ}}\right]$, while overripe tomatoes transition toward orange/brown tones $\left[20^{{}^{\circ}},40^{{}^{\circ}}\right)$. These hue-based differentiations form the fundamental basis for establishing a robust maturity classification model. The scoring formula based on color as the criterion is given in Equation 9.

**Table 2:** Threshold for judging tomato maturity based on color space.

Maturity stage	H (hue)
Unripe Tomato	$\left[50^{{}^{\circ}},100^{{}^{\circ}}\right)$
Ripe Tomato	$\left[0^{{}^{\circ}},20^{{}^{\circ}})\cup [330^{{}^{\circ}},360^{{}^{\circ}}\right]$
Overripe Tomato	$\left[20^{{}^{\circ}},40^{{}^{\circ}}\right)$

(9) $$S_{color}=\{\begin{array}{c} 1.0,\;\;\;ifH\in [0^{{}^{\circ}},20^{{}^{\circ}})\cup [330^{{}^{\circ}},360^{{}^{\circ}}]\\ h_{1}+c_{1}\cdot (40-H),\;\;ifH\in [20^{{}^{\circ}},40^{{}^{\circ}})\\ h_{2}+c_{2}\cdot (H-50),\;\;ifH\in [50^{{}^{\circ}},100^{{}^{\circ}}) \end{array}$$

$h_{1}$ is usually taken as 0.5, $h_{2}$ as 0.2, $c_{1}$ as 0.015, and $c_{2}$ as 0.01. Their values can be adjusted according to the actual situation.

##### 2.2.2.2 Tomato maturity judgment based on diameter size

This study developed a three-dimensional spatial positioning algorithm for tomatoes through the integrated operation of the GAE-YOLO model and ZED binocular cameras. The algorithm utilizes the width ( w) and height ( h) of the detection frame (in pixels) generated by GAE-YOLO, along with the depth perception capability of the ZED camera, to compute the three-dimensional coordinates (w,h,z) of tomatoes, where z denotes the actual distance (in millimeters) from the tomato to the camera. The system implementation is based on key parameters derived from camera calibration, including the focal length ( f) in pixels. The actual width ( $w_{real}$) and height ( $h_{real}$) of tomatoes are calculated using the following formulas given in Equations 10, 11.

(10) $$w_{real}=\frac{w\cdot z}{f_{w}}$$

(11) $$h_{real}=\frac{h\cdot z}{f_{h}}$$

where $f_{w}$ and $f_{h}$ represent the horizontal and vertical focal lengths of the camera (in pixels), respectively. Based on these parameters, the diameter of the tomato can be calculated using the formula given in Equation 12.

(12) $$d_{real}=\frac{(w_{real}+h_{real})\cdot z}{f_{w}+f_{h}}$$

It should be noted that the diameter-based maturity judgment thresholds for tomatoes require calibration according to specific cultivars, with the current system configured based on the empirically derived thresholds presented in **Table 3**. The scoring formula for using diameter as a maturity criterion is given in Equation 13.

**Table 3:** Threshold for judging tomato maturity based on diameter size.

Maturity stage	Diameter threshold (example: beefsteak tomato)
Unripe Tomato	(0,50)
Ripe Tomato	[50,70]
Overripe Tomato	(70,~)

(13) $$S_{size}=\{\begin{array}{c} s_{1}\cdot \frac{d_{real}}{unripe},\;if\;d_{real}<d_{unripe}\\ s_{1}+s_{2}\cdot \frac{d_{real}-d_{unripe}}{d_{ripe}-d_{unripe}},\;if\;d_{unripe}<d_{real}<d_{ripe}\\ 1.0-s_{3}\cdot \frac{d_{real}-d_{ripe}}{d_{overripe}-d_{ripe}},\;if\;d_{real}>d_{ripe} \end{array}$$

where $d_{unripe}$ denotes the maximum diameter of unripe tomatoes, $d_{ripe}$ represents the standard diameter of ripe tomatoes, and $d_{overripe}$ indicates the minimum diameter of overripe tomatoes. The weight coefficients are typically assigned as $s_{1}=0.3$, $s_{2}=0.7$, and $s_{3}=0.2\;$based on empirical validation.

##### 2.2.2.3 Tomato maturity judgment based on shape

This study presents a tomato maturity assessment method utilizing shape characteristics. The tomato size is first derived from the GAE-YOLO detection bounding box, followed by preliminary maturity estimation through aspect ratio calculation (Equation 14). While this approach demonstrates high computational efficiency, its accuracy is somewhat limited, making it particularly suitable for large-scale cultivation scenarios. The threshold for judging tomato shape maturity is presented in **Table 4**.

(14) $$r=\frac{w_{real}}{h_{real}}$$

**Table 4:** Threshold for judging tomato shape maturity.

Maturity stage	Aspect ratio range
Unripe Tomato	(0.9,1.1)
Ripe Tomato	(0.7,0.9]∪[1.1,1.3)
Overripe Tomato	(~,0.7]∪[1.3,~)

A high-precision contour analysis-based algorithm can be implemented to extract tomato ROIs (Regions of Interest) from GAE-YOLO detection frames. Following binarization processing, contours are extracted and the area-to-perimeter ratio is calculated. While this approach enables more accurate shape assessment for individual tomato plants, its application in large-scale cultivation scenarios is not recommended due to compromised computational efficiency.

The scoring formula for tomato maturity based on shape is defined in Equation 15.

(15) $$S_{shape}=\{\begin{array}{c} 1.0-p_{1}\cdot |r-1|,\;\;if\;|r-1|\leq 0.3\\ 0.7-p_{2}\cdot (|r-1|-0.3),\;\;if\;0.3<|r-1|\leq 0.7\;\;\\ 0.4-p_{3}\cdot (|r-1|-0.7),\;\;if|r-1\;|>0.7 \end{array}$$

For uniformly shaped immature or mature tomatoes, the aspect ratio typically falls within the range of [0.7, 1.3]. However, mature tomatoes may exhibit moderate aspect ratio deviations due to slight deformations. In contrast, overripe, diseased, or mechanically damaged tomatoes often demonstrate significant aspect ratio variations resulting from severe morphological changes. The weighting coefficients are empirically assigned as $p_{1}$ = 0.2, $p_{2}$ = 0.3, and $p_{3}$ = 0.4.

In summary, this study has constructed a tomato maturity assessment model that integrates multi-feature fusion of HSV color space, size, and shape characteristics, as formulated in Equation 16.

(16) $$RipenessScore=\omega_{c}\cdot S_{color}+\omega_{d}\cdot S_{size}+\omega_{s}\cdot S_{shape}$$

where $S_{color}$, $S_{size}$, $S_{shape}$ normalized scores for color, size, and shape features, $\omega_{c}$, $\omega_{d}$, $\omega_{s}$ corresponding weighting coefficients $\omega_{c}+\omega_{d}+\omega_{s}=1$.

All feature scores are normalized to the range (0,1), and the final maturity determination rule is mathematically formulated in Equation 17.

(17) $$RipenessScore=\{\begin{array}{c} immature,\;\;if\;RipenessScore<0.4\\ ripe,\;\;if\;0.4\leq RipenessScore\leq 0.7\\ overripe,\;\;if\;RipenessScore>0.7 \end{array}$$

In the tomato maturity assessment model, the weight distribution among HSV color space, size, and shape features must be adaptively adjusted based on both their discriminative contributions to maturity evaluation and practical application requirements.

#### 2.2.3 Method for estimating the yield of tomato plants

This study develops a tomato volume calculation method integrating GAE-YOLO detection with depth vision technology. The true tomato volume is calculated by combining depth information ( z) acquired from the ZED binocular camera and pre-calibrated focal length ( f) parameters with the detected width ( $w_{real}$) and height ( $h_{real}$). Under the idealized spherical assumption for tomato morphology, the volume of individual tomatoes is estimated using the formula provided in Equation 18.

(18) $$v=\frac{4}{3}\cdot \pi \cdot {\left(\frac{d_{real}}{2}\right)}^{3}$$

However, since tomatoes are not perfect spheres, an ellipticity correction factor k (typically k=0.85-0.95, as shown in Equation 19) must be introduced to adjust Equation 18:

(19) v=k·v

This study has developed a single-plant tomato yield estimation method based on 3D spatial clustering. The method employs tomato three-dimensional coordinates acquired by ZED cameras and implements the density-based DBSCAN (Bi et al., 2012) clustering algorithm to accurately associate fruits with individual plants. The core principle posits that density-connected samples belong to the same cluster, whereby fruits from the same plant exhibit spatial proximity (high-density regions), while fruits from different plants or isolated fruits demonstrate spatial separation (low-density regions).

The implementation is specified as follows: Given a tomato plant’s 3D coordinate point $p=(w_{real},h_{real},z)$ and neighborhood radius ϵ, its neighborhood is defined as Equation 20:

(20) $$N_{\epsilon }(p)=\{q\in dataset\;|\;dist(p,q)\leq \epsilon \}$$

where dist(p,q) denotes the Euclidean distance between points p and q, as shown in Equation 21. Points within this neighborhood are merged into the current cluster, ensuring density-reachability within clusters.

(21) $$dist(p,q)=\sqrt{{\left(w_{real{\_}_{p}}-w_{real{\_}_{q}}\right)}^{2}+{\left(h_{real_{p}}-h_{real_{q}}\right)}^{2}+{\left(z_{p}-z_{q}\right)}^{2}}$$

Therefore, the calculation formula of tomato yield per plant is given in Equation 22.

(22) $$n_{per\_accurate}=\omega \cdot \sum_{i}^{q}v_{i}$$

where ω denotes the density parameter, which needs to be determined according to specific tomato cultivars.

#### 2.2.4 Intelligent diagnosis of tomato foliar diseases based on large models

This study developed visualization software for intelligent tomato diagnosis and management to address challenges in leaf disease prevention and control during tomato cultivation. In current agricultural practice, the absence of professional guidance often leads to difficulties in accurately identifying disease symptoms (e.g., distinguishing yellow spots caused by leaf mold versus nutrient deficiency), resulting in improper control measures and subsequent losses. To overcome these issues, an innovative visual interactive interface was designed based on the PyQt6 framework, integrating core functional modules including maturity analysis, foliar disease identification, and prevention recommendations. Large model network APIs were implemented to enable real-time diagnosis on Jetson TX2 edge devices, ensuring smooth operation even with limited computational resources.

### 2.3 Evaluation indicators

This study selects precision rate, recall rate, mean average precision (mAP), frames per second (FPS), and GFLOPS as the performance metrics for evaluating deep learning models. The calculation formula for the evaluation indicators is as follows.

Precision (P): Measures the proportion of true positive predictions among all positive predictions made by the model. The formula is given in Equation 23.

(23) $$Precision=\;\frac{TP}{TP+FN}\times 100\%$$

Recall (R): Measures the proportion of true positive predictions among all actual positive instances. The formula is given in Equation 24.

(24) $$Recall=\frac{TP}{TP+FN}\times 100\%$$

where TP represents true positives (correctly detected targets), FP represents false positives (incorrectly detected targets), FN represents false negatives (missed targets).

Precision and recall values are used to construct the precision-recall curve (PR curve), with the area under this curve denoted as AP (Average Precision), as shown in Equation 25.

(25) $$AP=\int_{0}^{1}P(R)dR$$

The average of AP values across all categories in the dataset. The formula is given in Equation 26.

(26) $$mAP=\frac{\sum_{i=1}^{n}AP_{i}}{n}$$

where mAP@0.5 represents the mAP at an Intersection over Union (IoU) threshold of 0.5, commonly used to evaluate the model’s localization accuracy.

T denotes the detection time per image, while FPS represents the number of images processed per second, as shown in Equation 27.

(27) $$FPS=\frac{1}{T}$$

In addition, GFLOPs reflects the computational complexity of the model, serving as an important metric for measuring computational efficiency.

### 2.4 Experimental details

The Model training and development configuration for this study is configured as follows: CPU: Intel (R) Core (TM) i9-13900H, RAM: 32GB, GPU: NVIDIA GeForce RTX 4060 with 8GB of video memory. The framework environment is built on PyTorch 2.5.1, with Python version 3.9.21 (running on Windows 11), CUDA version 12.1, and CUDNN version 9.0.0. All experiments were conducted with uniform parameter settings, and the specific hyperparameters are listed in **Table 5**.

**Table 5:** Experimental hyperparameters.

Hyperparameter	Value
Initial Learning Rate	0.01
Momentum	0.937
Batch Size	16
Image Size	640×640
Training Epochs	300
Seeds	[42, 123, 2023]

The edge computing device TX2 is configured with: NVIDIA Parker series SoC (64-bit ARM architecture), CPU (dual-core Denver 2), GPU (256-core NVIDIA Pascal architecture GP10B), 8GB LPDDR4 memory, running on Ubuntu 18.04 LTS. To ensure maximum computational throughput, the module was operated in its default MAX-N performance mode. For power profiling, collecting data over a continuous 10-mins window under sustained workload, establishing a reliable power consumption baseline.

The GAE-YOLO model trained on RTX 4060 was converted into an optimized inference engine for TX2 using TensorRT, significantly improving execution speed while reducing resource consumption. The PyTorch model was first exported to ONNX format to ensure full operator compatibility with TensorRT. The ONNX model was then loaded and optimized using TensorRT tools on TX2 to generate the final engine file. By strategically distributing computational tasks across TX2’s GPU, DLA, and CPU, maximum resource utilization was achieved.

## 3 Results and analysis

The experimental results of the intelligent tomato model developed in this study are systematically presented across three key aspects: (1) tomato fruit detection performance, (2) foliar disease identification accuracy, and (3) integrated diagnostic capabilities. To ensure statistical robustness, our proposed model was trained and evaluated over three independent runs with different random seeds. Accordingly, a statistical reporting protocol has been adopted: the performance metrics for our model are reported as the mean ± standard deviation, while results for comparative models are from a single representative run.

**Figure 9:**
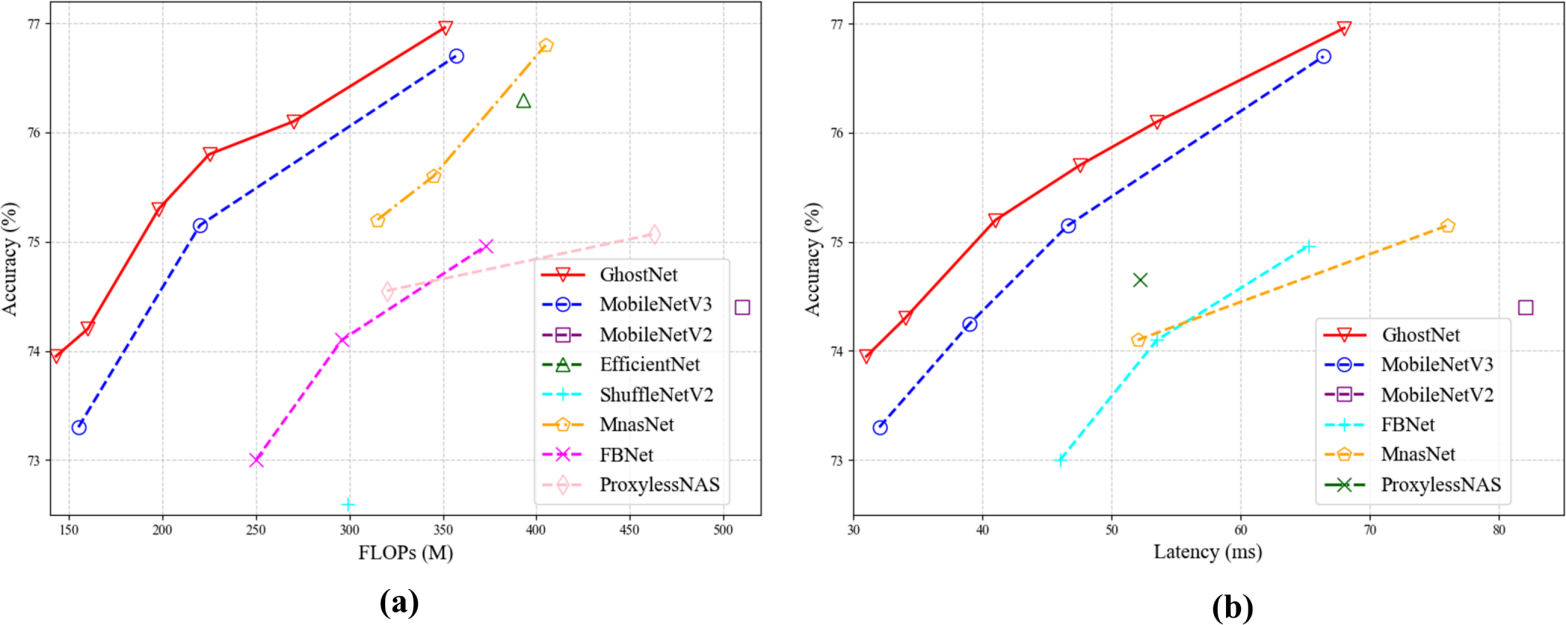
Comparison of lightweight models **(a)** Comparison of lightweight models in flops. **(b)** Comparison of lightweight models in latency.

To validate the advantages of GhostConv’s lightweight architecture (Han et al., 2020), performed systematic experiments on the ImageNet classification task. The results demonstrate a positive correlation between computational resource utilization and model accuracy in these compact networks, thereby conclusively verifying its superior performance characteristics. **Figure 9** presents a comprehensive comparison between GhostNet and existing lightweight models in terms of FLOPs and inference latency. A critical finding of the comparative analysis is that GhostNet emerges as the more efficient architecture, achieving approximately 0.5% higher top-1 accuracy than MobileNetV3 under equivalent latency while also requiring less time to reach a comparable performance level. Moreover, evaluation across different complexity scales confirms that this performance advantage is consistently maintained, demonstrating the architecture’s robust superiority.

### 3.1 Tomato detection results and analysis

This study establishes model lightweighting as the primary objective and conducts a systematic evaluation of mainstream lightweight architectures for tomato detection tasks. Using the standard YOLOv11 as the baseline model, the most representative lightweight network architectures were selected for comparative analysis. The YOLOv11 backbone was modified by implementing depthwise separable convolution and its derivative architectures, including MobileNetV3 and EfficientNet. Through controlled experiments, the performance of each model was rigorously compared and analyzed across key dimensions: precision, recall, mAP, GFLOPS. The architectural exploration led to a key revelation: the GhostConv-based modification proved to be an optimal strategy, successfully maintaining high detection accuracy (mAP@50 = 93.5%) while reducing computational complexity by 12.6% to 5.5 GFLOPS compared to the baseline. Importantly, under comparable computational constraints, GAE-YOLO consistently surpassed all other benchmarked models, an outcome that solidifies its status as the most effective solution evaluated. These findings conclusively validate the technical advantages of GAE-YOLO for agricultural edge computing applications. The detailed results are presented in **Table 6**.

**Table 6:** Comparison of modified by GhostConv with other lightweight improvement models (RTX 4060, FP32, 1080p).

Model	P	R	mAP@50	mAP@95	GFLOPS
Baseline	0.880	0.886	0.933	0.639	6.3
Backbone modified by DC	0.854	0.794	0.901	0.587	5.4
Backbone modified by MobileNetv3	0.865	0.821	0.887	0.603	5.5
Backbone modified by Efficient	0.873	0.833	0.911	0.601	5.5
Baseline modified by GhostConv (ours)	0.871 ± 0.09%	0.878 ± 0.10%	0.925 ± 0.09%	0.620 ± 0.13%	5.5 ± 0.37%

Recent advancements in YOLO series improvements have primarily focused on attention mechanisms and loss function optimization. However, these approaches often prioritize theoretical algorithmic enhancements while overlooking the critical need for lightweight designs in practical implementations. To bridge this gap, this study introduces the innovative GAE-YOLO model, which achieves an optimal balance between lightweight architecture and high detection accuracy through carefully engineered modifications. Specifically, two parameter-efficient enhancement strategies are incorporated: AReLU and E-IoU loss function, both designed to improve performance without increasing model parameters. To validate the efficacy of the proposed model, systematic comparative experiments were conducted (see **Table 7**). The results demonstrate that GAE-YOLO maintains high detection accuracy (mAP@50 = 93.2%) while achieving real-time processing at 10.2 FPS on Jetson TX2 edge devices. Notably, an FPS threshold exceeding 10 is required to ensure smooth target detection performance on the TX2 platform.

**Table 7:** Comparison of GAE-YOLO with other advanced modified models.

Model	P	R	mAP@50	mAP@95	GFLOPS	FPS	FPS(TX2)
Baseline	0.880	0.886	0.931	0.639	6.3	83	7.7
Baseline+CBAM	0.881	0.884	0.933	0.641	7.9	81	7.5
Baseline+CBAM+GS	0.882	0.881	0.931	0.643	7.6	85	8.1
Baseline+ iRMB	0.891	0.883	0.932	0.649	8.2	72	6.1
Baseline+GS+AReLU (ours)	0.889 ± 0.12%	0.884 ± 0.11%	0.933 ± 0.13%	0.648 ± 0.16%	5.5 ± 0.43%	96 ± 2.3%	10.2 ± 2.4%

FPS is measured under: (RTX 4060, FP32, 1080p) for FSP; (Jetson TX2, PyTorch, MAX-N, 1080p) for FPS (TX2).

**Table 8** presents a systematic ablation study of the GAE-YOLO model, evaluating the individual contributions and synergistic effects of each enhancement module through controlled experiments. The experimental design follows a progressive integration strategy, sequentially incorporating three core modules: GhostConv, E-IoU, and AReLU (where “✓” denotes module inclusion). As the foundational lightweight component, GhostConv achieves 0.925 mAP@50 and 0.620 mAP@95 while maintaining high-speed performance of 96 FPS (GPU 4060) and 10.2 FPS (TX2). The addition of the E-IoU loss function yields a 2.7% improvement in mAP@95, demonstrating significant enhancement in bounding box regression precision. When integrated with the AReLU activation function, mAP@95 further increases by 4.0%, validating the efficacy of the adaptive feature activation mechanism. The complete model combining all three modules achieves optimal performance (0.933 mAP@50 and 0.648 mAP@95), representing improvements of 0.8% and 4.5% over the baseline, respectively, without additional computational overhead. Consistent GFLOPS and FPS metrics across all experimental groups confirm that the proposed enhancements achieve performance gains at minimal computational cost. On the TX2 edge device, the full model maintains real-time processing at 10.2 FPS under a 1080p resolution without TensorRT optimization, demonstrating the baseline performance and satisfying deployment requirements for practical agricultural applications.

**Table 8:** Ablation experiment results of the GAE-YOLO model.

GhostConv	E-IoU	AReLu	mAP@50	mAP@95	GFLOPS	FPS	FPS(TX2)
✓			0.925	0.620	5.5	96	10.2
✓	✓		0.928	0.637	5.5	96	10.2
✓		✓	0.931	0.645	5.5	96	10.2
✓	✓	✓	0.933 ± 0.13%	0.648 ± 0.16%	5.5 ± 0.43%	96 ± 2.3%	10.2 ± 2.4%

FPS is measured under: (RTX 4060, FP32, 1080p) for FSP, (Jetson TX2, PyTorch, MAX-N, 1080p) for FPS (TX2).

**Figure 10:**
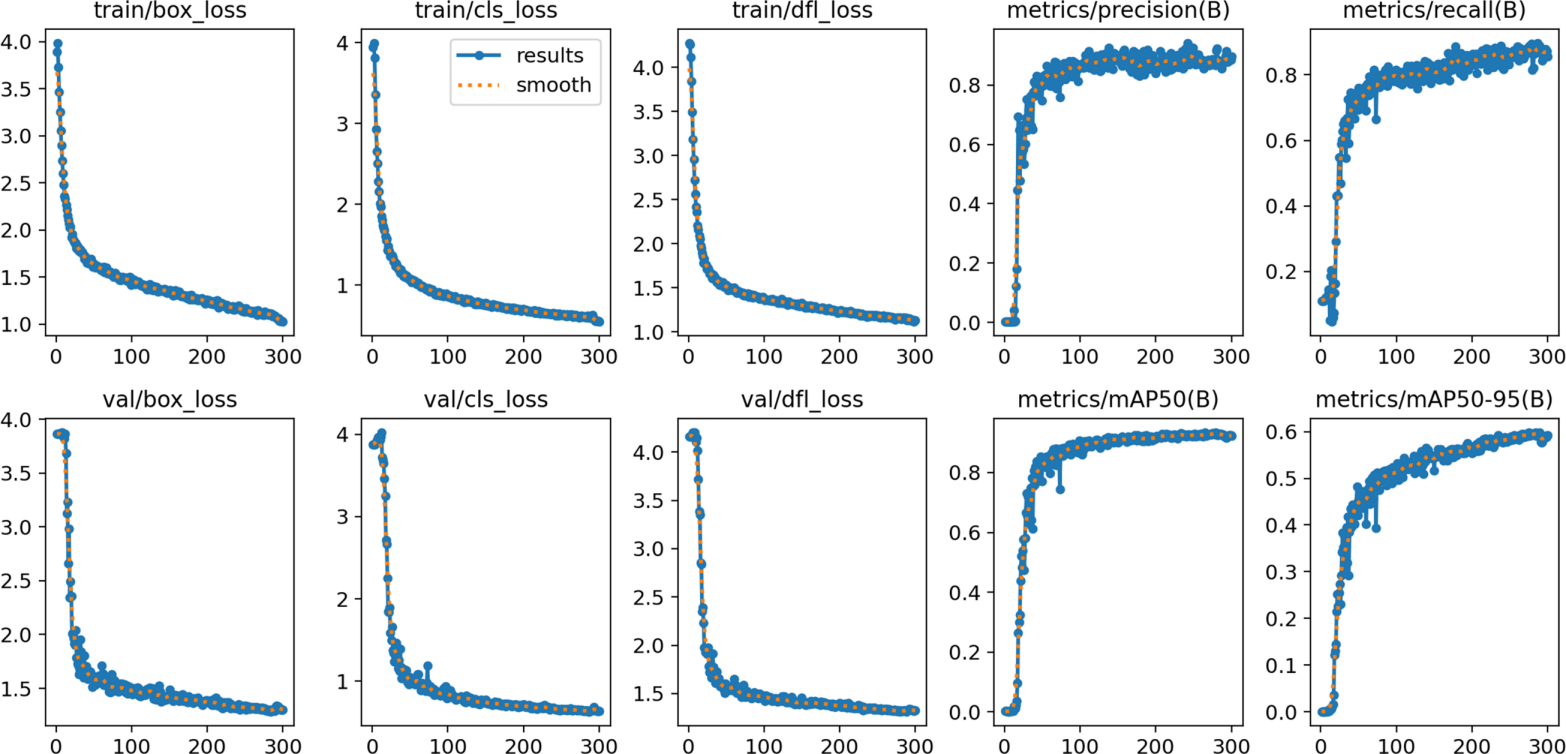
Visualization results of the GAE-YOLO training process (RTX 4060, FP32, 1080p).

**Figure 10** illustrates the training dynamics of the GAE-YOLO model, exhibiting optimal convergence behavior and learning efficiency. During the initial training phase (0–50 epochs), a rapid decrease in loss function values is observed alongside a corresponding sharp increase in mAP@50, demonstrating the model’s effective capture of essential tomato target features. In subsequent training stages (50–250 epochs), the loss curve slope progressively diminishes while mAP improvements stabilize, consistent with established deep learning optimization patterns. Notably, all metrics reach equilibrium during the final 250–300 epoch period, with this synchronous convergence validating the model’s architectural soundness. These results confirm that GAE-YOLO simultaneously avoids overfitting risks while achieving target detection accuracy objectives.

**Figure 11:**
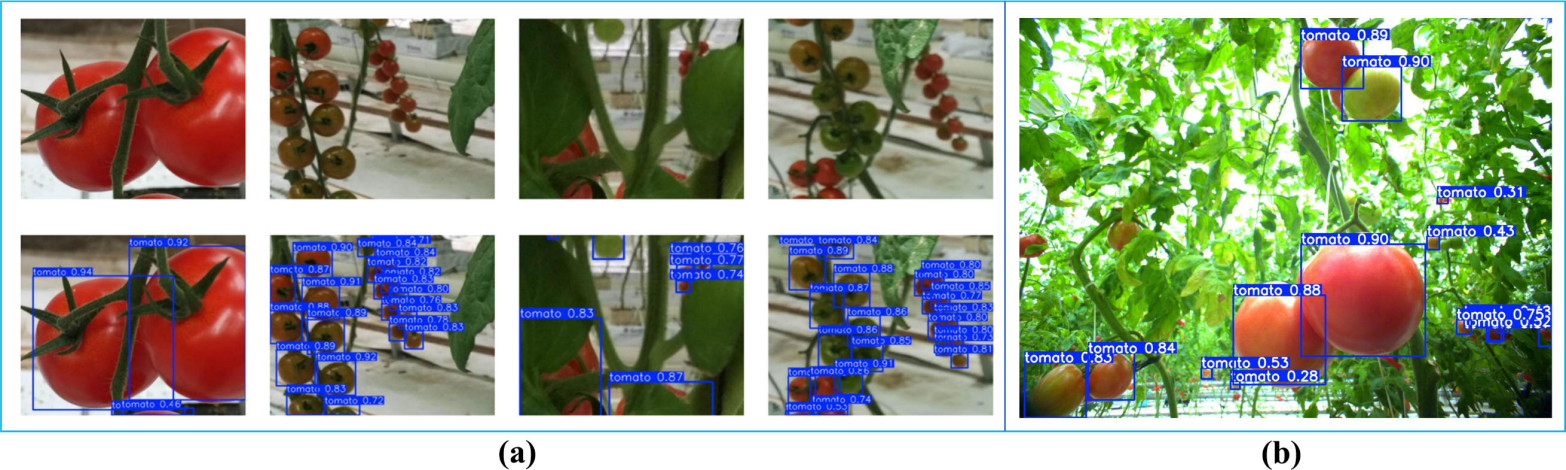
Visualization results of GAE-YOLO tomato detection. **(a)** Standardized test set. **(b)** Real greenhouse scenario.

**Figure 11** demonstrates the superior performance of the GAE-YOLO model in tomato detection tasks. Specifically, **Figure 11a** displays the visual detection results on the standardized test set, while **Figure 11b** showcases its application performance in real greenhouse environments, where high detection accuracy is maintained despite complex background interference. Through visual analysis, two primary error sources are identified: (1) missed detections due to severe foliage occlusion, and (2) false positives involving unripe tomatoes with leaf-like coloration. These errors predominantly occur in high-density fruit clusters with minimal inter-fruit spacing. These results validate not only the model’s detection capability under controlled conditions but also its practical performance in real-world agricultural scenarios.

### 3.2 Detection results and analysis of tomato foliar diseases

**Table 9** presents the performance evaluation results of the GAE-YOLO model for tomato leaf disease detection, demonstrating its capabilities across different disease categories through multiple metrics. The model achieves an overall precision of 0.756, recall of 0.725, mAP@50 of 0.724, and mAP@95 of 0.492, indicating strong detection performance. Healthy leaves show the best results with 0.967 precision, 0.99 recall, and 0.995 mAP@95, confirming accurate health/disease discrimination. Bacterial spot and late blight achieve mAP@50 scores of 0.747 and 0.921 respectively, reflecting good recognition of visually distinctive diseases, while early blight shows higher recall (0.827) than precision (0.753), suggesting some false positives. Target spot maintains balanced metrics (P = 0.619, R = 0.667), indicating stable feature learning, whereas leaf mold and black spot perform poorly (black spot mAP@95: 0.185) due to challenging visual characteristics like irregular shapes and low color contrast. Notably, the significant performance gap between mAP@50 and mAP@95 (average difference: 0.232) highlights ongoing challenges in precise lesion boundary localization.

**Table 9:** Detection results of tomato foliar diseases (RTX 4060, FP32, 1080p).

Calss	P ( ≤|±0.12%|)	R ( ≤|±0.11%|)	mAP@50 ( ≤|±0.13%|)	mAP@95 ( ≤|±0.16%|)
all	0.756	0.725	0.724	0.492
Bacterial Spot	0.934	0.750	0.747	0.531
Early_Blight	0.753	0.827	0.783	0.446
Healthy	0.967	0.99	0.995	0.929
Late_blight	0.823	0.862	0.921	0.749
Leaf Mold	0.564	0.571	0.497	0.251
Target_Spot	0.619	0.667	0.679	0.352
Black spot	0.396	0.396	0.445	0.185

The significant performance variation across disease categories, as detailed in **Table 9**, warrants further analysis. Diseases like Black Spot and Leaf Mold present particular challenges primarily due to their subtle and irregular visual characteristics. In early stages, Black Spot lesions are small, dark, and lack a defined shape, making them difficult to distinguish from shadows or soil splashes. Similarly, Leaf Mold often manifests as diffuse, chlorotic areas with low color contrast against the healthy green leaf background, confounding the model. These challenges are compounded by two factors: (1) Intra-class variability, where the appearance of a single disease can vary significantly, and (2) Inter-class similarity, where early symptoms of different diseases can be visually analogous. The substantial gap between the mAP@50 and mAP@95 metrics further underscores the model’s difficulty in achieving precise pixel-level localization for these complex, non-uniform lesions. This difficulty is reflected in the large mAP@50–95 gap, highlighting a struggle with precise lesion localization. To address this, future work should prioritize enhanced multi-scale feature fusion to capture both minute spots and broad discolorations, alongside class-balanced training strategies to directly counteract data imbalance. Incorporating attention mechanisms also presents a promising path for improving focus on these subtle, critical features.

**Figure 12:**
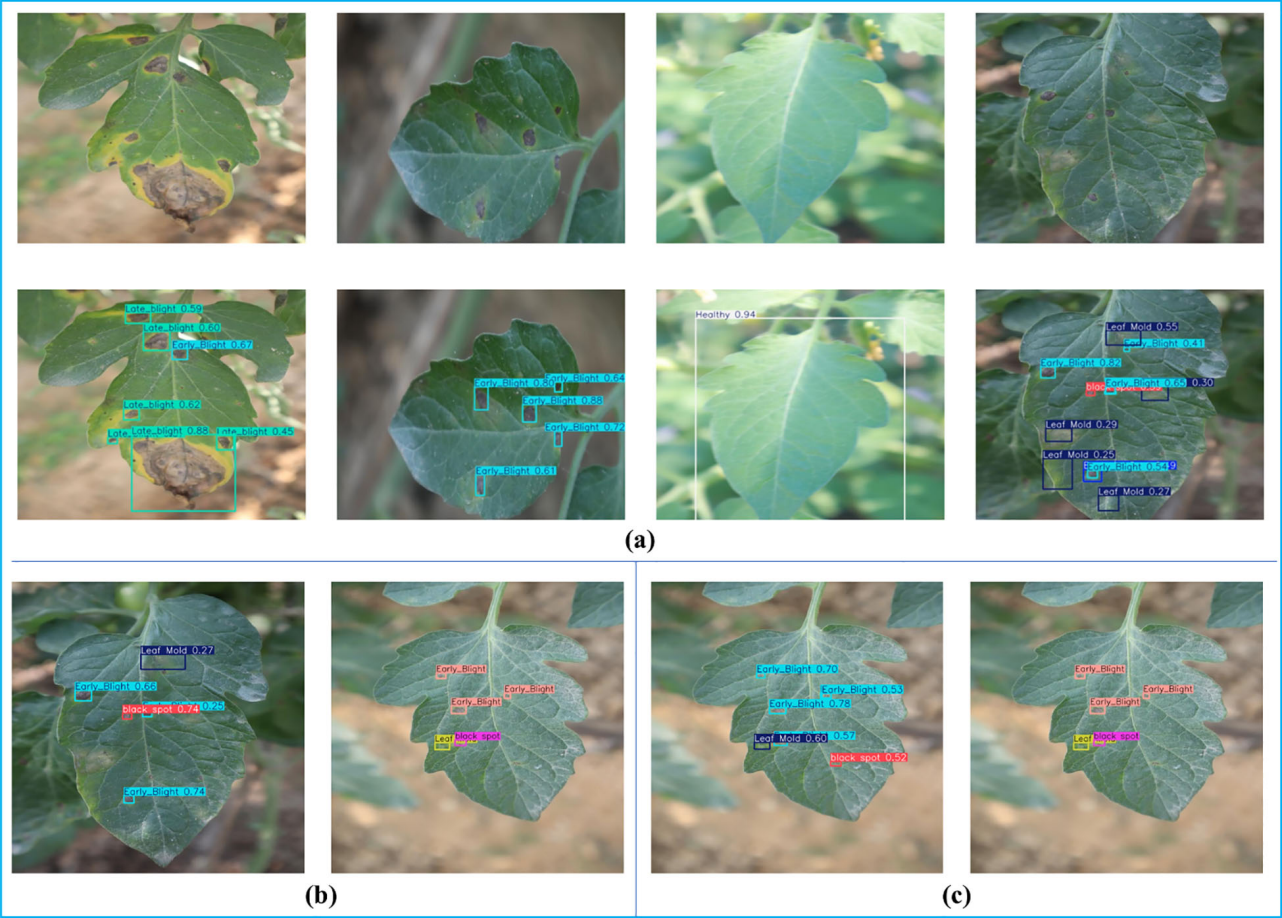
Visual detection results and error case analysis of tomato leaf diseases based on GAE-YOLO. **(a)** Successful detection cases under challenging conditions. **(b, c)** Representative error cases contrasting model predictions (left) with ground-truth annotations (right).

**Figure 12** demonstrates the performance of the GAE-YOLO model in tomato leaf disease detection tasks, showcasing both its robust capabilities and characteristic failure modes. **Figure 12a** shows the model accurately identifying and localizing multiple disease instances under challenging conditions, confirming its strong baseline performance. **Figures 12b, c** present representative error cases to visually contextualize the quantitative analysis discussed in the text: each panel contrasts the model’s prediction (left) with the ground-truth annotation (right).

**Figure 13:**
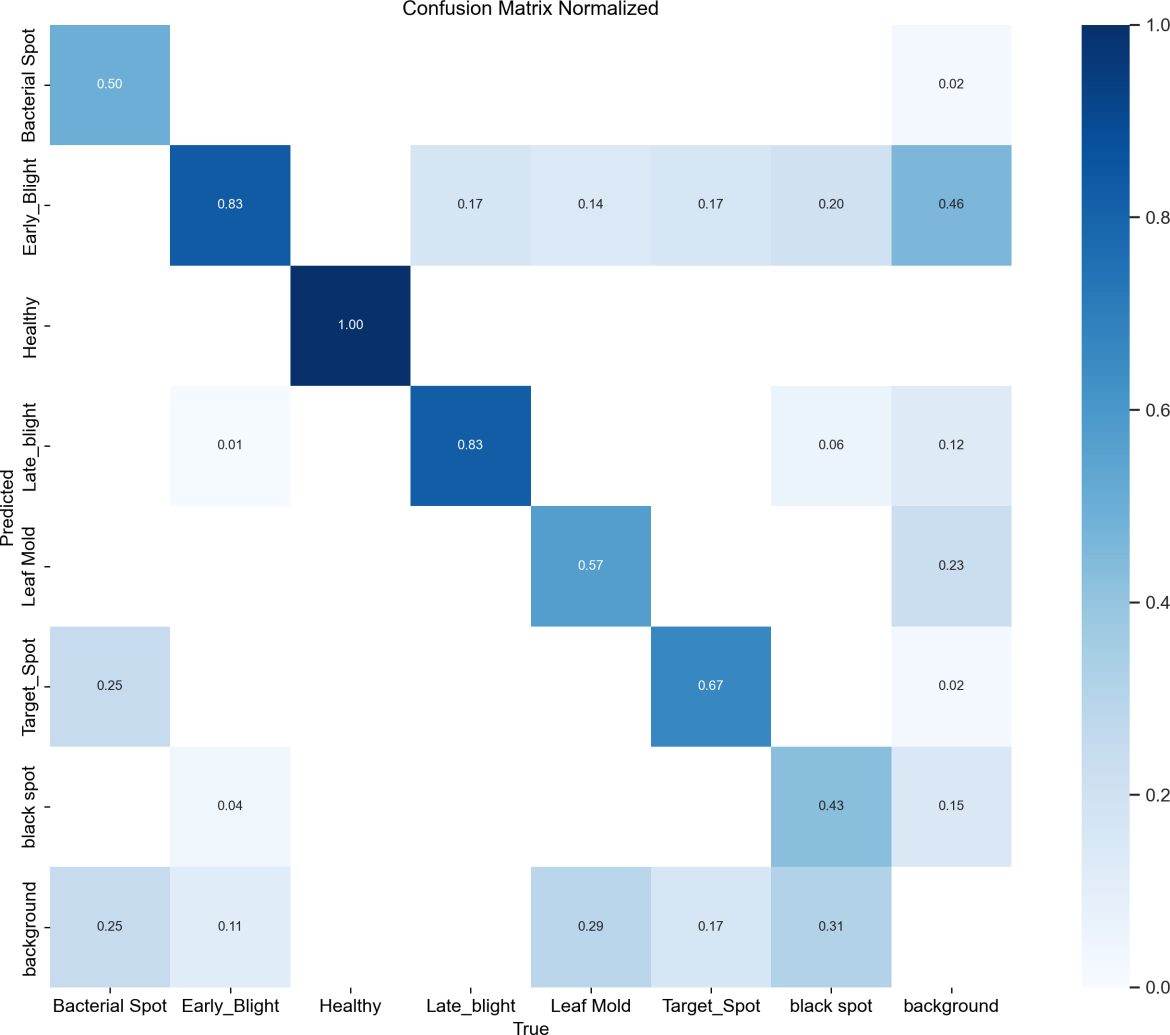
Confusion matrix of tomato leaf disease detection (RTX 4060, FP32, 1080p).

**Figure 13** presents the classification performance of the model for tomato leaf disease detection through a normalized confusion matrix. The matrix reveals that healthy leaves are identified with the highest accuracy (main diagonal value: 0.99). However, disease category recognition, particularly for early blight demonstrates significant challenges, with only 46% correct identification and 83% misclassification as healthy leaves. Other disease categories (e.g., leaf mold and black spot) exhibit varying confusion levels, primarily due to visual similarity in early-stage symptoms. These results highlight the model’s current limitations in detecting subtle disease features, providing critical insights for subsequent optimization of early disease identification capabilities. The observed performance gap, particularly for minority classes such as black spot and leaf mold, is primarily attributed to class imbalance within the dataset. To mitigate this issue and the associated risk of overfitting, several targeted strategies can be implemented during training: (1) a class-weighted loss function can be employed to increase the penalty for misclassifying minority classes, thereby directing greater attention to them; (2) specialized data augmentation techniques—such as PCA color augmentation, random rotation, and noise injection—can be preferentially applied to these under-represented classes to increase their effective sample size and feature diversity.

### 3.3 Results and analysis of tomato maturity discrimination

**Figure 14:**
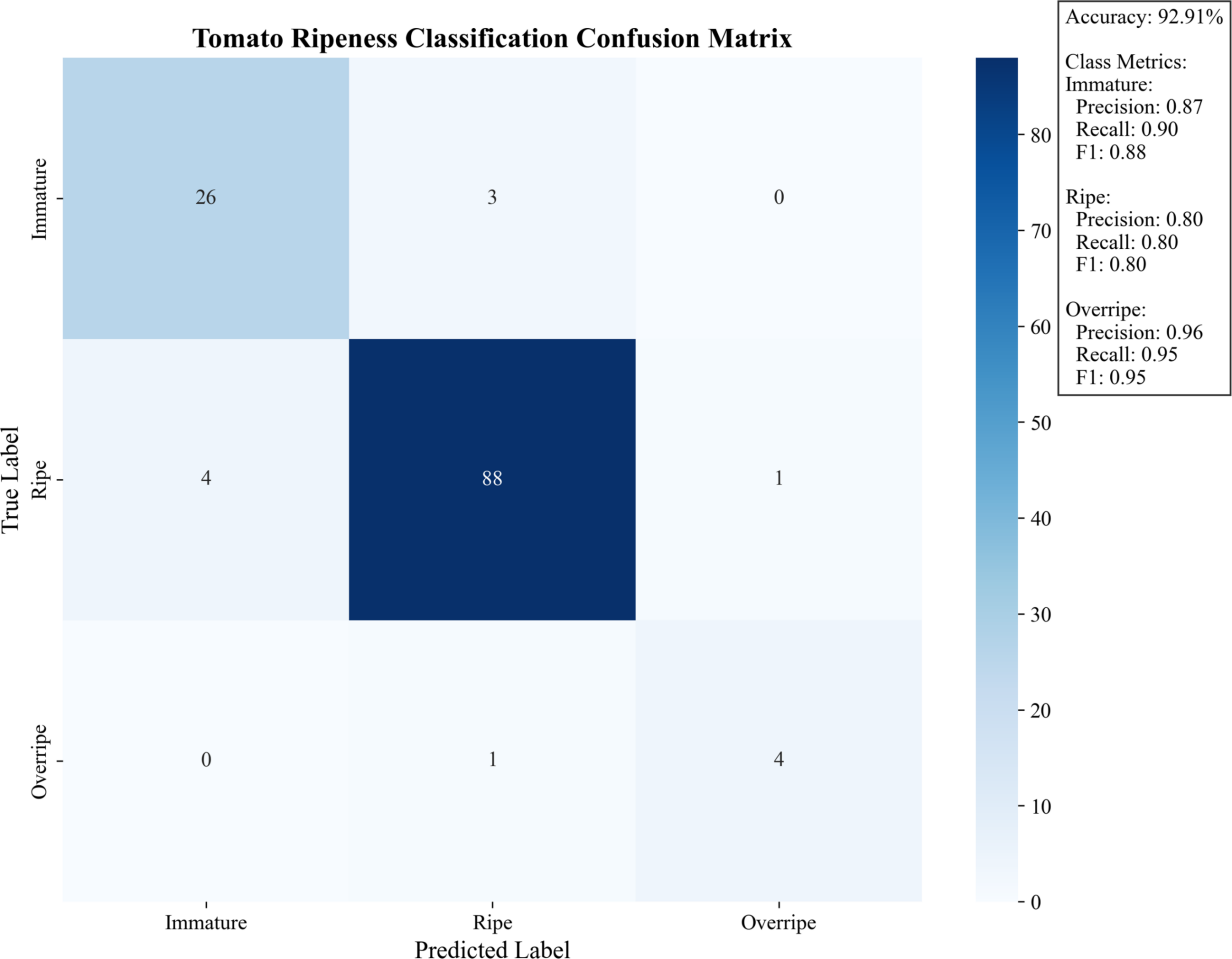
Confusion matrix for judging tomato ripeness (Jetson TX2, PyTorch, MAX-N, 1080p).

**Figure 14** presents the tomato maturity classification confusion matrix, demonstrating the model’s performance in maturity discrimination by visualizing the correspondence between predicted labels (immature, mature, overripe) and ground truth labels. The model achieves an overall accuracy of 92.91%, with particularly strong performance in overripe category identification (F1-score: 0.95) and relatively weaker performance in mature category discrimination (F1-score: 0.80). Detailed error analysis reveals that among 88 immature samples, 1 was misclassified as mature, while mature samples showed 4 misclassifications as immature and 1 as overripe, with only 1 overripe sample misclassified as mature. This error distribution indicates persistent challenges in distinguishing adjacent maturity stages (particularly immature-to-mature transitions), with most misclassifications occurring during color transition phases, though the model demonstrates high reliability in overripe state identification (precision/recall: 0.95). It should be noted that tomato maturity assessment currently lacks unified standards and authoritative benchmark datasets, with practical harvesting still relying on subjective judgment. To ensure experimental rigor, this study implemented random selection of 15 tomato plants as samples and consensus evaluations from 5 experienced agricultural growers. While this methodology is widely recognized in the industry, its inherent subjectivity reflects broader challenges in agricultural visual inspection. The achieved performance is particularly significant given this challenging context, as it establishes a critical quantitative baseline for future research. This experimental design not only mirrors real-world assessment criteria but also establishes a critical baseline for developing more objective maturity quantification systems.

### 3.4 Estimated results and analysis of tomato production

This study employs tomato fruit count as the primary yield assessment metric, based on two key considerations: (1) the substantial density variations across tomato varieties make quantity-based evaluation more robust against varietal characteristic biases, and (2) fruit count per plant represents the most intuitive and rapidly verifiable yield parameter in agricultural management practices. This approach not only fulfills standardized evaluation requirements during algorithm development but also establishes a reliable foundation for practical weight conversion applications.

**Figure 15:**
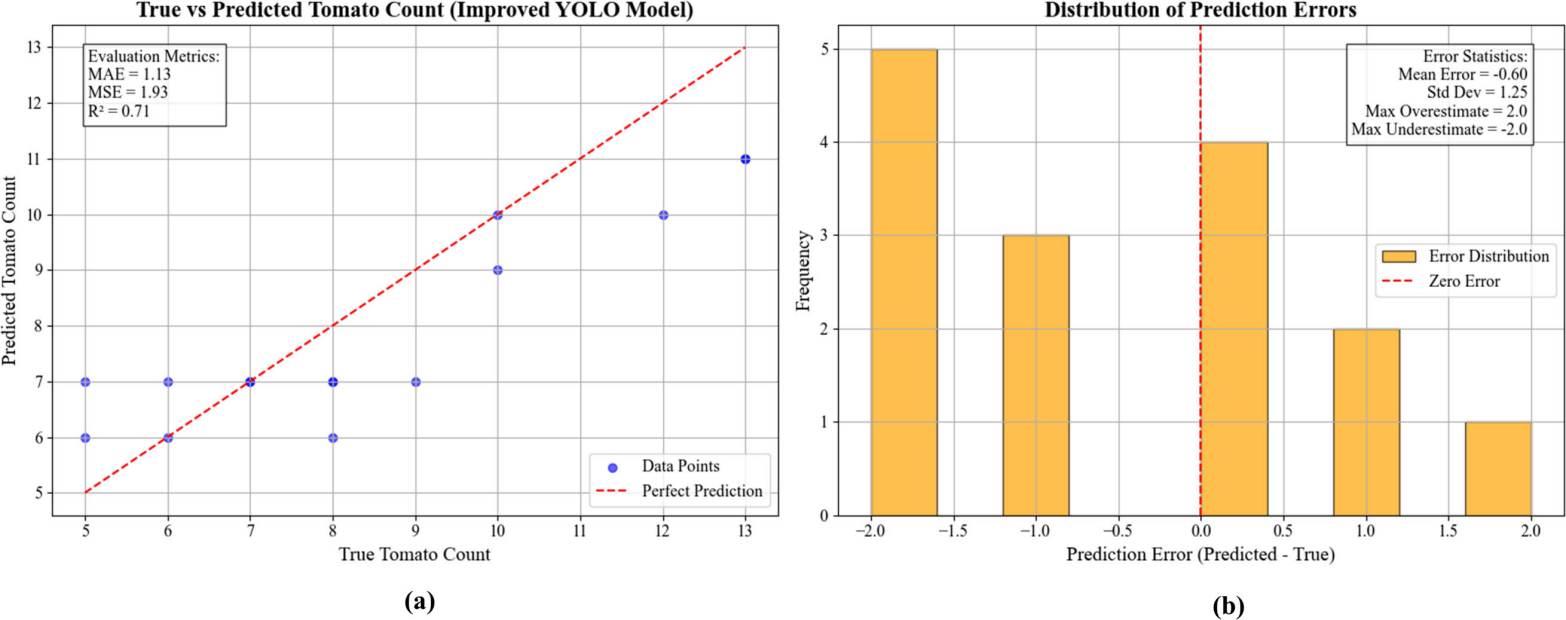
Tomato count and error distribution graph and error distribution histogram (Jetson TX2, PyTorch, MAX-N, 1080p). **(a)** Scatter plot of true versus predicted tomato counts with model metrics. **(b)** Histogram showing the distribution of prediction errors.

**Figure 15** presents the performance evaluation of the GAE-YOLO model for tomato counting tasks through dual-perspective analysis. The actual vs. predicted scatter plot demonstrates strong linear correlation (R²=0.71), confirming the model’s capability to capture tomato quantity variations. However, most data points distribute below the ideal prediction reference line, revealing a systematic underestimation tendency that becomes pronounced in high-count regions (actual>8) with maximum underestimation reaching 2. Quantitative metrics (MAE = 1.13, MSE = 1.93) confirm the model’s practical prediction accuracy. The error distribution histogram shows approximately normal characteristics (μ=-0.60, σ=1.25), with 68% predictions within ±1.25 of actual counts. While extreme over/under-estimation values show symmetric distribution (± 2.0), the overall negative bias confirms conservative prediction behavior. Although slight systematic underestimation persists, primarily caused by leaf occlusion and fruit overlap, 94% of errors remain within ±2 counts, satisfying agricultural yield prediction requirements.

### 3.5 Analysis of memory and power consumption

A comprehensive analysis of resource utilization is crucial for assessing the feasibility of deploying intelligent systems in real-world agricultural scenarios, where computational resources and power are often constrained. The system achieves a detection accuracy of 93.5% mAP@50 under a high-precision configuration, with a processing speed of 10.2 FPS. Under the optimized configuration, the system achieved a real-time processing speed of 27 FPS on the TX2 edge device, with a corresponding per-frame latency of 33 ms. This low latency ensures prompt responsiveness for robotic control, while the frame rate satisfies the throughput requirement for continuous monitoring.

The runtime memory footprint of the complete system comprising the Jetson TX2 module, the ZED stereo camera operating at 720p resolution, and the TensorRT-optimized GAE-YOLO model was rigorously profiled. Using the integrated tegrastats system monitoring tool over multiple operational cycles, a peak memory consumption of 5.1 GB was recorded. This measurement confirms that the system operates comfortably within the 8 GB memory budget of the Jetson TX2, leaving approximately 2.9 GB of available headroom. This surplus memory is sufficient to accommodate other essential robotic processes, such as simultaneous data logging, path planning algorithms, or communication modules, without risking system instability.

Power Consumption and Deployment Feasibility. The operational power budget is a key determinant for sustained field deployment. Based on the documented thermal design power of the Jetson TX2 module (ranging from 7.5W to 15W), the total system power draw is estimated to be between 10W and 13W when operating in the default MAX-N performance mode. This efficient power profile enables long-duration operation using standard, commercially available power sources. Several viable options are identified: A standard 12V,10Ah lithium polymer battery can theoretically power the system for 9 to 12 hours of continuous operation. High-capacity mobile power banks supporting 12V output via power delivery trigger modules offer a portable and flexible power solution. Configurations such as 4S2P (8 cells), providing a nominal 14.8V and high capacity, represent another robust and rechargeable option.

To quantitatively evaluate the inference speed achieved under different deployment scenarios, a comparative analysis was conducted across the development platform and the edge device under various optimization settings. The results are summarized in **Table 10**.

**Table 10:** Comparative inference performance across different hardware and optimization configurations.

Configuration	Hardware	Framework	Power mode	Precision	Resolution	FPS
GAE-YOLO(GPU)	RTX4060	Pytorch	N/A	FP32	1080P	96
GAE-YOLO(TX2)	Jetson TX2	Pytorch	MAX-N	FP32	1080P	10.2
GAE-YOLO(TX2)	Jetson TX2	TensorRT	MAX-N	FP16	720P	27

### 3.6 Design of tomato smart detection and diagnosis software

**Figure 16:**
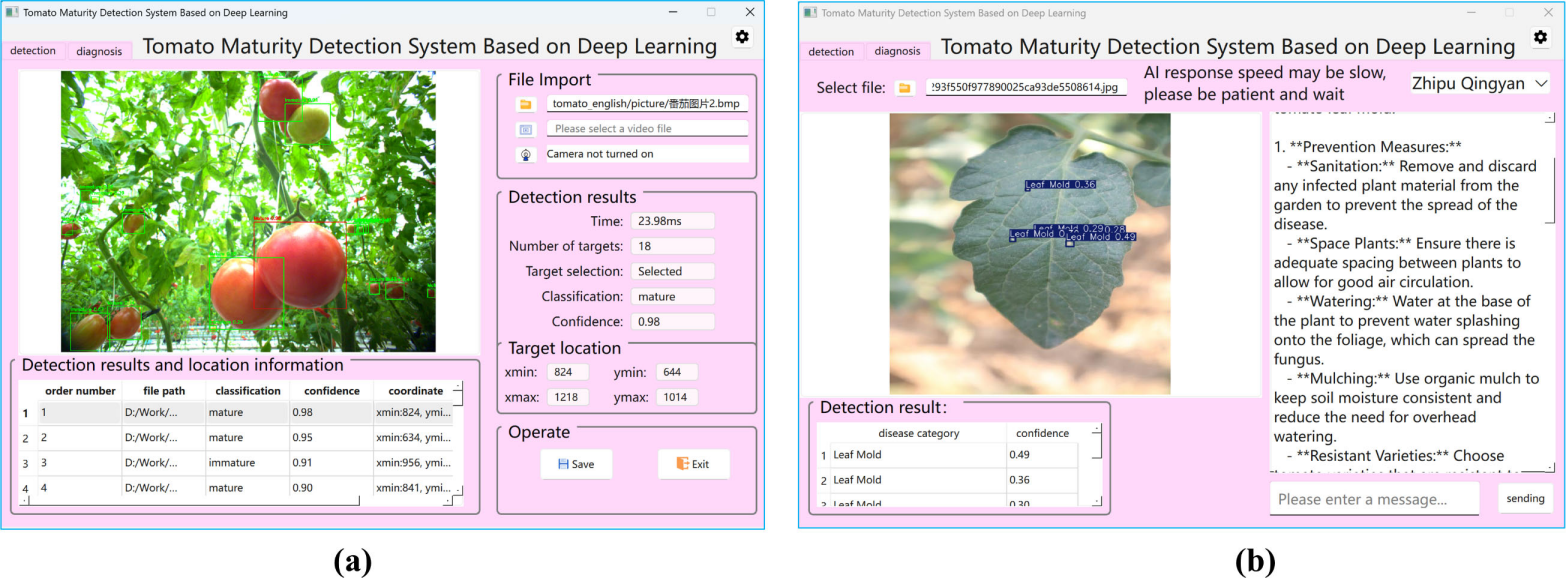
Tomato smart detection and diagnosis software. **(a)** Tomato detection and growth monitoring module. **(b)** Disease diagnosis and control module.

This study developed intelligent tomato cultivation management software based on the PyQt6 framework, with its visual interface design presented in **Figure 16**. The software features a modular architecture comprising two core functional modules: (1) a tomato detection and growth monitoring module (**Figure 16a**) integrating fruit localization, maturity analysis, and yield prediction functions; and (2) a disease diagnosis and control module (**Figure 16b**) enabling disease spot detection, disease classification, and treatment recommendation generation. Compared with conventional agricultural management systems, this software employs an intuitive graphical interface that transforms complex AI algorithms into simplified button operations, offering a cost-effective digital solution for small-to-medium greenhouse operations.

## 4 Discussion

The GAE-YOLO model and intelligent agricultural management system developed in this study demonstrate significant innovations and practical value through three key aspects. First, at the algorithmic level, the proposed model overcomes the limitations of conventional object detection models in agricultural applications by incorporating GhostConv modules to reduce parameters and enhance inference speed, effectively addressing the computational constraints of edge devices. A critical finding from the systematic architectural comparison was the superior performance of GhostConv over other lightweight backbones like MobileNetV3 and EfficientNet. This advantage is attributed to GhostConv’s fundamental operating principle: it generates a portion of its feature maps through efficient, linear transformations rather than relying solely on costly dense convolutions. This mechanism preserves richer spatial context and feature channel information compared to the extreme channel compression of depthwise separable convolution used in MobileNetV3. For the specific challenge of distinguishing tomatoes from similarly colored leaves and complex backgrounds, this retained contextual information proves more critical than simply maximizing theoretical FLOPs reduction, thereby providing a more favorable accuracy-efficiency trade-off. The novel AReLU activation function and E-IoU loss function further enable the model to maintain high accuracy in complex agricultural environments. Second, regarding system integration, this work pioneers the deep integration of binocular vision and edge computing, where the coordinated operation of ZED cameras and Jetson TX2 achieves precise tomato 3D localization while the intelligent agricultural advisory system provides effective disease prevention solutions. Additionally, the established tomato maturity assessment and per-plant yield estimation models overcome the functional limitations of traditional agricultural management systems, forming a complete detection-analysis-decision closed loop. Compared with existing studies, this system offers three distinct advantages: (1) first implementation of a complete detection-to-management pipeline, (2) balanced real-time performance and high accuracy, and (3) significantly reduced operational complexity through intuitive visual design. Notwithstanding these contributions, it is imperative to address the limitations pertaining to dataset scope and model generalization. While the dataset is representative and rigorous augmentation strategies were employed to mitigate overfitting, its finite size inherently limits the model’s exposure to the full spectrum of variability encountered in unstructured agricultural environments. Consequently, the model’s performance may be susceptible to scenarios not well-represented in the training data, such as unprecedented disease manifestations, extreme abiotic stresses, or unique cultivar characteristics. This study proactively combated overfitting through advanced regularization and data augmentation, yet the pursuit of robust generalization remains an ongoing challenge in agricultural computer vision. In this context, several specific limitations should be noted: (1) model generalization requires larger sample sizes, (2) performance under extreme lighting conditions needs improvement, and (3) multi-cultivar adaptability requires further validation. Addressing these challenges will constitute the primary focus of future research.

## 5 Conclusion

This study developed an intelligent tomato management system based on the GAE-YOLO algorithm, which addresses key challenges in agricultural production through algorithmic innovations and technological integration. The primary contributions include: (1) the GAE-YOLO lightweight model, achieving real-time tomato detection at 10.2 FPS on Jetson TX2 with 93.3% mAP@50 in real-world agricultural environments; (2) the first integrated 3D tomato detection system combining binocular vision and edge computing; (3) a standardized tomato maturity assessment system and yield estimation model, establishing a foundation for future research; and (4) visual management software incorporating large model technology for tomato disease control. These advancements not only demonstrate the value of technological innovation but also provide scalable solutions for smart agriculture development. The significance of this research is twofold: theoretically, it proposes a lightweight model design methodology tailored for agricultural applications; practically, it delivers a cost-effective smart agriculture system, contributing substantially to precision agriculture and sustainable development goals.

## Data Availability

The datasets presented in this study can be found in online repositories. The names of the repository/repositories and accession number(s) can be found below: The data and code supporting this study are publicly available at GitHub under the following links: https://github.com/NSSCk/GAE-YOLO.
